# Endocrine-Disrupting Pesticides as Drivers of Human Disease: Mechanistic Toxicology and Life-Course Health Effects

**DOI:** 10.3390/ijms27156928

**Published:** 2026-08-01

**Authors:** Nour El-Hoda Zidan, Tarek Alshaal, Nevien Elhawat, Osama Elhamalawy, Farag Malhat, Fawzy Eissa

**Affiliations:** 1 Pesticides Chemistry and Toxicology Department, Faculty of Agriculture, Kafrelsheikh University, Kafr El-Sheikh 33516, Egypt; nourelhoda_az@hotmail.com; 2 Department of Food Biotechnology, Albert Kázmér Mosonmagyaróvár Faculty, Széchenyi István University, 9026 Győr, Hungary; 3 Environment and Bio-agriculture Department, Faculty of Agriculture, Al-Azhar University, Cairo 11884, Egypt; osamaelhamalawy@yahoo.com (O.E.); fawzy.eissa@yahoo.com (F.E.); 4 Central Agricultural Pesticide Laboratory, Pesticide Residues and Environmental Pollution Department, Agricultural Research Center, Giza 12618, Egypt; farag_malhat@yahoo.com

**Keywords:** pesticide exposure, reproductive health, endocrine-related cancers, metabolic disorders (obesity; diabetes), thyroid hormone disruption, neurodevelopmental outcomes (ADHD; ASD)

## Abstract

Endocrine-disrupting pesticides (EDPs) are environmental toxicants capable of perturbing hormonal homeostasis through multiple molecular and cellular mechanisms. Growing evidence indicates that these compounds contribute to a broad spectrum of adverse health outcomes extending beyond classical endocrine dysfunction. This review critically synthesizes current knowledge on the toxicological mechanisms of EDPs and evaluates epidemiological evidence linking exposure to human disease. Mechanistically, EDPs act through modulation of nuclear hormone receptors, disruption of membrane-associated signaling pathways, interference with hormone synthesis, metabolism, and transport, induction of oxidative stress and mitochondrial dysfunction, and epigenetic reprogramming. These molecular events converge on shared biological pathways that affect multiple organ systems and life stages. Human and experimental evidence associates EDP exposure with reproductive dysfunction, endocrine-related cancers, metabolic disorders, thyroid abnormalities, and neurodevelopmental impairments. Particular concern surrounds exposure during critical windows of susceptibility, especially prenatal development and early childhood, when endocrine systems are highly vulnerable to disruption and developmental programming. Across disease endpoints, recurring mechanisms, including endocrine receptor perturbation, oxidative stress, inflammation, and epigenetic alterations, support a unifying toxicological framework linking diverse adverse outcomes. Despite substantial progress, important uncertainties remain regarding chronic low-dose exposure, non-monotonic dose–response relationships, cumulative effects of pesticide mixtures, and the translation of mechanistic findings into human risk assessment. Future research should integrate repeated biomonitoring, advanced mixture modeling, mechanistic biomarkers, and multi-omics approaches within longitudinal life-course studies. Improved integration of toxicological and epidemiological evidence will strengthen causal inference, refine hazard characterization, and support more protective regulatory strategies for reducing the human health burden associated with endocrine-disrupting pesticides worldwide.

## 1. Introduction

Pesticides have become indispensable tools in modern agriculture, enabling increased crop yield and food security for growing global populations. Yet their widespread use has raised profound concerns regarding unintended consequences for human health. Among these concerns, the capacity of certain pesticides to disrupt endocrine function has emerged as a critical area of scientific inquiry demanding urgent attention. Endocrine-disrupting chemicals (EDCs) represent a broad and critically important group of exogenous substances or mixtures that alter the function(s) of the endocrine system and consequently cause adverse health effects in an intact organism, its progeny, or (sub)populations [1,2]. Within this context, endocrine-disrupting pesticides (EDPs) constitute a diverse class of agrochemicals that interfere with hormonal homeostasis through multiple interconnected mechanisms, including nuclear receptor interactions, non-genomic signaling disruption, alterations in hormone synthesis and metabolism, epigenetic modifications, and oxidative stress induction [1,2,3]. These compounds, ranging from legacy organochlorine insecticides such as DDT and its metabolites to currently used herbicides like glyphosate and atrazine, can mimic or antagonize endogenous hormones, thereby perturbing the delicate biochemical processes that govern development, reproduction, and metabolic regulation [4,5].

The health consequences associated with EDP exposure are extensive and affect individuals across the entire lifespan, from prenatal development through adulthood. Epidemiological evidence has consistently linked these compounds to reproductive disorders in both sexes, including impaired fecundability, prolonged time-to-pregnancy, reduced semen quality characterized by decreased sperm concentration and motility, and developmental anomalies in offspring such as cryptorchidism and hypospadias [6,7,8,9]. Women exposed to organochlorine pesticides demonstrate elevated risks of endometriosis and polycystic ovary syndrome, along with compromised ovarian reserve, while men exhibit disruptions of the hypothalamic–pituitary–gonadal axis and increased sperm DNA fragmentation [10,11,12]. Furthermore, robust associations have been documented between pesticide exposure and endocrine-related malignancies, particularly cancers of the prostate, breast, and testis, with biologically plausible mechanisms involving hormonal modulation, oxidative stress, and genotoxicity [13,14,15]. Beyond reproductive and oncological outcomes, metabolic disorders represent another major category of health effects associated with EDP exposure. Obesity, type 2 diabetes, insulin resistance, and metabolic syndrome have been consistently associated with organochlorine and pyrethroid pesticide exposure across diverse populations and study designs [16,17,18,19]. Prenatal exposure to DDT and related compounds has been linked to increased adiposity in childhood and adulthood, suggesting that developmental windows represent periods of heightened vulnerability [20,21]. Additionally, thyroid dysfunction, including altered circulating hormone levels and increased thyroid cancer risk, and neurodevelopmental disorders such as attention deficit hyperactivity disorder and autism spectrum disorder have been linked to prenatal and childhood pesticide exposure through mechanisms involving disruption of thyroid hormone signaling and neurotransmitter systems [22,23,24,25]. Beyond prenatal and early childhood stages, vulnerability extends across key physiological transitions throughout the life course: during puberty and adolescence, EDP exposure disrupts gonadotropins, estradiol, and IGF-1, leading to delayed sexual maturation and menstrual irregularities [6,10], while during perimenopause, menopause, and aging, endocrine senescence heightens susceptibility to pesticide-induced thyroid dysfunction [24] and hormone-sensitive malignancies, including postmenopausal breast and ovarian cancers [13]. Although endocrine-disrupting chemicals have been widely reviewed, analyses focused specifically on pesticides remain fragmented across chemical classes, mechanistic pathways, or single disease categories. Many previous reviews emphasize experimental toxicology or selected clinical outcomes, particularly reproductive toxicity, without systematically integrating the broader spectrum of human endocrine-sensitive disorders. This review synthesizes current understanding of the mechanisms through which EDPs exert their effects and critically examines the epidemiological evidence connecting these exposures to adverse health outcomes across multiple organ systems. This review integrates mechanistic toxicology with human epidemiological findings across reproductive disorders, endocrine-related cancers, metabolic dysfunction, thyroid disease, and neurodevelopmental outcomes. By adopting a life-course perspective and highlighting cross-cutting themes such as low-dose mixture effects, sex-specific susceptibility, vulnerable developmental windows, and transgenerational implications, this review aims to provide a more comprehensive and conceptually integrated account of the multifaceted threats posed by EDPs. Through this integrative approach, this article seeks not only to summarize existing knowledge but also to identify shared biological pathways and recurring methodological gaps that are critical for improving causal inference, guiding future research, and informing more protective regulatory policy.

## 2. Modes of Action of EDPs

**Figure 1:**
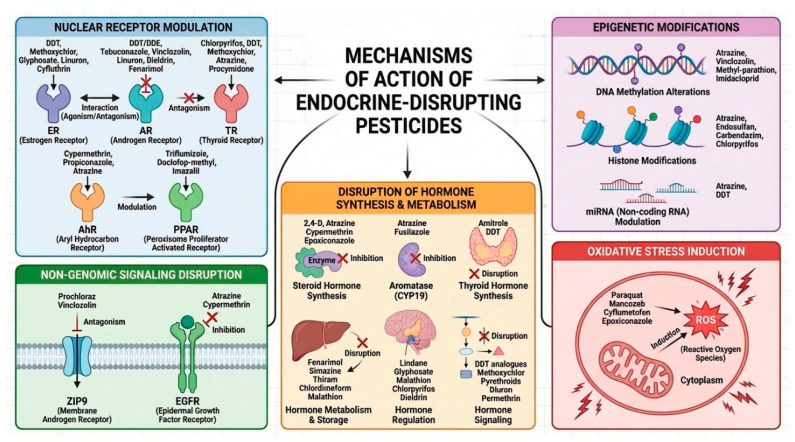
Mechanisms of action of endocrine-disrupting pesticides.

EDPs interfere with hormonal homeostasis through multiple mechanisms (Table S1). The following subsections summarize major, well-characterized pathways by which EDPs exert their endocrine-disrupting effects (Figure 1).

### 2.1. Nuclear Receptor Interactions

EDPs can act as agonists or antagonists of nuclear hormone receptors, disrupting normal hormonal signaling. For example, DDT and methoxychlor mimic endogenous estrogens, activating estrogen receptors (ERα/ERβ) and leading to upregulation of estrogen-responsive genes such as *Pgr*, *Ccnd1*, and *Cyp19a1*, ultimately resulting in feminization effects [1,2]. Methoxychlor’s metabolite HPTE has potent agonistic activity for ESR1 but antagonistic activity for ESR2 [4]. Glyphosate enhances ERα phosphorylation and mimics estradiol in breast cancer cells [5]. Conversely, compounds like DDE and vinclozolin act as androgen receptor antagonists, disrupting androgen-mediated gene expression and reproductive development [2,26]. Linuron and triazole fungicides further inhibit androgen signaling [27,28]. EDPs such as chlorpyrifos and pyrethroids also interfere with thyroid receptors, affecting neurodevelopment [29,30,31]. Other nuclear receptors targeted include PPARγ and AhR, as activated by triflumizole and cypermethrin, respectively, affecting adipogenesis and detoxification [32,33,34,35].

### 2.2. Non-Genomic Signaling Disruption

Some EDPs act through rapid, non-genomic pathways involving membrane-bound receptors. Prochloraz and vinclozolin antagonize ZIP9, a membrane androgen receptor, thereby blocking testosterone-induced zinc influx and apoptotic signaling in prostate cells [36]. Atrazine and cypermethrin interfere with EGFR and its downstream MAPK signaling, impairing cell proliferation and differentiation [37]. Additionally, pesticides can disrupt rapid non-genomic pathways by binding to other membrane-bound steroid targets, including the G-protein coupled estrogen receptor (GPER) and membrane progesterone receptors (mPRs), as well as by directly modulating membrane ion channels such as the sperm-specific, steroid-activated CatSper Ca^2+^ channel, which alters calcium influx and compromises cell function [2].

### 2.3. Disruption of Hormone Synthesis, Metabolism, and Transport

EDPs can impair hormone synthesis by targeting steroidogenic enzymes and cholesterol transport. The herbicide 2,4-D reduces testosterone by interfering with cholesterol delivery to mitochondria in Leydig cells [38,39]. Atrazine enhances estrogen and androgen production in adrenal cells [40], while cypermethrin alters steroidogenic enzyme expression and elevates estrogen, cortisol, and aldosterone levels [41]. Mancozeb, ziram, and endosulfan suppress testosterone synthesis and enzyme expression [42,43]. Epoxiconazole disrupts steroid biosynthesis by inhibiting CYP51A1 [44,45]. Additionally, atrazine and flusilazole inhibit aromatase (CYP19), reducing estrogen synthesis [2,46]. Thyroid hormone biosynthesis is impaired by amitrole and DDT, which affect thyroglobulin transcription and iodide uptake [47,48]. Some compounds, including fenarimol, simazine, thiram, and malathion, disrupt hormone metabolism and storage [49,50,51,52]. Others such as lindane, glyphosate, and chlorpyrifos interfere with hormone regulation by inhibiting cholinesterase activity or affecting neurotransmitters [53,54]. Pesticides like methoxychlor and permethrin disrupt hormone signaling pathways, leading to reproductive and developmental abnormalities [3,55].

### 2.4. Epigenetic Modifications

EDPs induce heritable changes in gene expression via epigenetic mechanisms such as DNA methylation and histone modification. Atrazine and vinclozolin alter DNA methylation in sperm, resulting in transgenerational reproductive effects [56]. Methyl-parathion and o,p’-DDT cause global hypomethylation and promoter-specific hypermethylation [57,58]. Atrazine also affects histone H3K4me3 patterns in sperm [59], while endosulfan increases HDAC and DNMT expression [60]. Carbendazim alters H3K27me3 marks and DNA methylation in testes [61]. DDT and atrazine modify miRNA expression in hormone-sensitive cells and embryonic tissues [62,63].

### 2.5. Oxidative Stress Induction

Many EDPs induce oxidative stress, generating reactive oxygen species (ROS) that damage cellular components and hormone-producing tissues. Paraquat and mancozeb promote ROS accumulation, disrupting redox balance and impairing fertility [25,64]. Specifically, EDP exposure weakens key enzymatic antioxidant defenses, including superoxide dismutase (SOD), catalase (CAT), and glutathione peroxidase (GPx), while depleting cellular non-enzymatic antioxidants such as reduced glutathione (GSH) and total thiol groups [25,64]. Consequently, unmitigated ROS accumulation drives lipid peroxidation, evidenced by elevated malondialdehyde (MDA) levels, as well as protein carbonylation and DNA damage [45,64,65]. Cyflumetofen triggers apoptosis through oxidative stress in hormone-producing cells [66]. Epoxiconazole induces oxidative DNA and protein damage in developing tissues [45,65]. These redox disturbances can compromise hormone synthesis and signaling, especially during development. The key mechanisms of endocrine disruption, providing representative pesticide examples and their affected pathways are summarized in Table S1.

## 3. Health Impacts of EDPs

The disruption of hormonal balance by EDPs has been linked to a wide array of health problems across the lifespan.

### 3.1. Reproductive Health Issues

Several human studies have evaluated associations between pesticide exposure and a broad spectrum of adverse reproductive outcomes in both women and men (Table S2). In women, prospective cohort and case–control studies consistently link exposure to organochlorine and other pesticides with impaired fecundability, prolonged time-to-pregnancy, infertility, and ovarian dysfunction. Elevated concentrations of p,p’-DDE, HCB, β-HCH, and mirex have been associated with increased risks of endometriosis and polycystic ovary syndrome, along with reduced anti-Müllerian hormone levels, altered gonadotropins, and disrupted estradiol signaling, reflecting compromised ovarian reserve and endocrine dysregulation [6,10,67,68,69,70,71,72].

**Figure 2:**
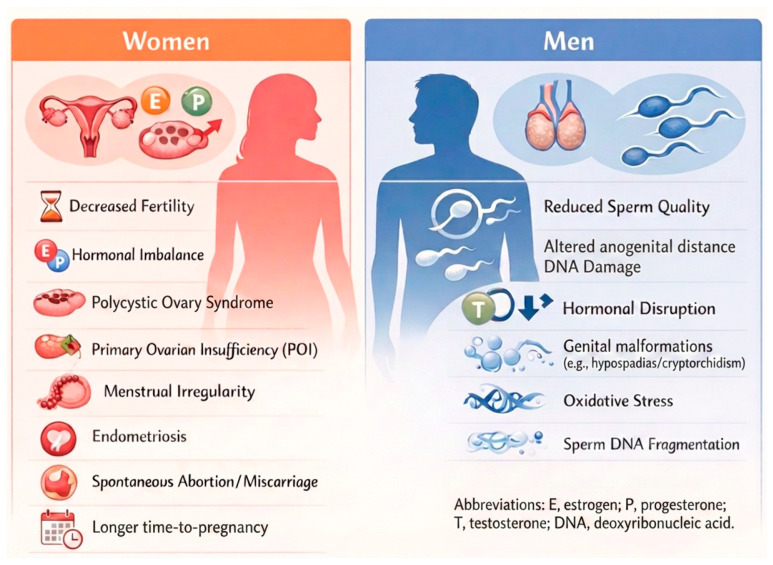
Reproductive health effects of pesticide exposure in women and men.

Urinary biomarkers of nonpersistent pesticides, including organophosphate and pyrethroid metabolites, have also been associated with infertility, primary ovarian insufficiency, and adverse hormone profiles, suggesting that both persistent and nonpersistent pesticides may adversely affect female reproductive function [73]. Moreover, cross-sectional studies further indicate that pesticide exposure is linked to menstrual irregularities, reduced gonadotropins during adolescence, and elevated risks of spontaneous pregnancy loss among occupationally exposed women, suggesting susceptibility of the female reproductive system throughout the life course [74,75,76]. In men, numerous cross-sectional, cohort, and retrospective studies have shown that occupational or environmental exposure to pesticides is linked to lower semen quality, including decreased sperm concentration, motility, vitality, and increased abnormal morphology [7,8,12,77,78]. Several studies further report pesticide-related disruptions of the hypothalamic–pituitary–gonadal axis, with altered levels of testosterone, estradiol, FSH, LH, SHBG, and free androgen indices, particularly in relation to exposure to DDT, DDE, and HCB [79,80,81]. Importantly, emerging evidence extends beyond conventional semen parameters, demonstrating increased sperm DNA fragmentation and defective chromatin decondensation among exposed men, highlighting the significance of genomic integrity as a critical endpoint in pesticide-related male infertility [11,12]. Additionally, prenatal exposure has been linked to altered reproductive development in male offspring, including changes in anogenital distance, increased risks of hypospadias and cryptorchidism, and long-term hormonal alterations [9,82,83,84,85]. A schematic overview is provided in Figure 2.

Mechanisms linking pesticide exposure to poor reproductive health include hormone signaling disruption and direct tissue damage. Several pesticides are known as endocrine disruptors, acting as estrogenic or anti-androgenic agonists or antagonists, leading to menstrual cycle irregularities, reduced fertility, and increased miscarriage risks [86]. They can also harm reproductive tissues and cause oxidative stress and DNA damage in reproductive cells, resulting in developmental issues and birth defects [87]. Moreover, prenatal exposure may trigger gene expression alterations related to reproductive development, causing lasting negative effects on reproductive health in later life [88].

### 3.2. Endocrine-Related Cancers

#### 3.2.1. Testicular Cancer

Epidemiological evidence suggests that pesticide exposure may contribute to the development of testicular germ cell tumors (TGCTs) by disrupting endocrine homeostasis and interfering with fetal testicular development (Table 1). To ensure a balanced evaluation and account for potential publication bias, Table 1 explicitly includes studies reporting null, non-significant, or inverse associations alongside positive findings such as registry-based cohort studies finding no overall TGCT risk from parental occupational pesticide exposure or biobanked prenatal organochlorine exposure [89,90]. Many pesticides, including organochlorines, act as EDCs that mimic or block natural hormones. Specifically, they alter androgen and estrogen signaling crucial for in utero masculinization [14,15]. This interference disrupts the differentiation of primordial germ cells, potentially leading to the persistence of germ cell neoplasia in situ (GCNIS), the accepted precursor lesion for TGCT [89,91].

Beyond hormonal mimicry, pesticides may induce oxidative stress and epigenetic modifications within the fetal testicular tissue. These mechanisms promote genomic instability and aberrant cell proliferation, further implicating them in TGCT pathogenesis [92]. Despite these plausible biological pathways, epidemiological findings remain inconsistent due to exposure misclassification and small sample sizes. To clarify whether and how pesticide-induced endocrine disruption impacts TGCT risk, future research must integrate prospective biomonitoring of maternal samples with advanced mixture analysis and molecular investigations of epigenetic changes [14,15].

#### 3.2.2. Prostate Cancer

A growing body of epidemiological research suggests that occupational and environmental exposure to pesticides is associated with an increased risk of prostate cancer, particularly among farmers, pesticide applicators, and manufacturing workers [93,94,95,106]. Large cohort analyses from the Agricultural Health Study (Table 1) have repeatedly reported elevated risks of total and especially aggressive prostate cancer with higher cumulative exposure to specific insecticides, notably organophosphate organodithioates such as fonofos, terbufos, malathion, dimethoate, and the organochlorine aldrin [94,95]. Crucially, epidemiological evidence distinguishes aggressive (clinically significant, high-grade, or distant/metastatic) prostate cancer from indolent disease, indicating that organodithioate organophosphates (fonofos, terbufos, malathion, dimethoate), carbaryl (with a 30-year lag), and aldrin are preferentially associated with aggressive phenotypes [94,95,98]. Mechanistically, experimental models suggest this enhanced aggressiveness and malignancy are linked to differential androgen receptor (AR) dysregulation, non-genomic membrane AR (ZIP9) antagonism that impairs apoptotic signaling, activation of EGFR/MAPK pathways, and persistent oxidative stress that induces genomic instability [36,37,94], whereas non-genomic membrane AR (ZIP9) antagonism specifically characterizes exposure to certain fungicides rather than these organophosphate compounds. The associations between pesticide exposure and prostate cancer are biologically supported by mechanisms involving endocrine disruption, oxidative stress, and genotoxicity; specifically, certain organochlorines and organophosphates can act as androgen receptor agonists or antagonists, altering hormonal homeostasis critical to prostate cell regulation, while others induce DNA damage through the generation of reactive oxygen species or inhibit DNA repair enzymes [94,107,108]. Several case–control studies in Canadian and U.S. farming populations suggested that prolonged exposure to certain pesticides may increase prostate cancer risk. The reported association appeared stronger at higher lifetime exposure levels, particularly for compounds such as DDT, lindane, simazine, carbaryl, malathion, endosulfan, and 2,4-D [93]. Meta-analyses and systematic reviews combining data from cohort and case–control studies have generally reported a small but statistically significant increase in cancer risk among agricultural workers and individuals exposed to pesticides. The overall relative risk was typically estimated between 1.1 and 1.3, while stronger associations were observed in highly exposed groups and in studies using more detailed exposure assessments [106,107,108]. Elevated risks have also been reported among pesticide manufacturing workers, particularly in plants producing phenoxy herbicides contaminated with dioxins and furans, which are potent immunotoxicants and endocrine disruptors [106], and among farmers in several regional cohorts and case–control studies [93,107]. Importantly, Table 1 also reflects a substantial body of null evidence that must be considered alongside positive findings: the large USA prospective cohort found no clear overall association between pesticide use and total prostate cancer risk [94], a French case–control study found no positive association between any organochlorine pesticide measured in periprostatic adipose tissue and aggressive prostate cancer [97], and USA prospective cohort analysis of carbaryl found no clear association with total or aggressive prostate cancer in unlagged analyses [98]. These null findings indicate that pesticide-related prostate cancer risk is neither universal nor consistent across all compounds, populations, or disease subtypes, and that the current evidence supports only a probable rather than definitive causal association. Collectively, these findings support a probable association between chronic pesticide exposure and prostate cancer, driven by complex interactions between hormonal modulation and cellular damage, especially for certain insecticide and herbicide classes and in genetically or familially susceptible men [94,107,108,109].

#### 3.2.3. Breast Cancer

Several epidemiological studies conducted across different populations and study designs have suggested an association between pesticide exposure and breast cancer, particularly for certain pesticides and exposure patterns (Table 1). Importantly, risks and mechanisms vary across disease subtypes and menopausal stages: while overall workplace pesticide exposure showed no uniform link when stratified by ER/PR hormone receptor status [99], specific organophosphate exposures (chlorpyrifos, terbufos) and dietary pesticide mixtures are significantly linked to premenopausal and postmenopausal breast cancer subtypes, respectively [13,102]. Furthermore, regional observational studies report that chronic exposure to herbicides like glyphosate, atrazine, and 2,4-D is associated with higher odds of lymph node metastasis [104]. Mechanistically, preclinical and in vitro studies indicate that EDPs may promote tumor malignancy not only through ERα phosphorylation and aromatase (CYP19) modulation in hormone-dependent cells [2,5], but also via non-hormonal driver pathways including microRNA dysregulation, AhR activation, nitrosative stress, systemic Th1/Th17 cytokine suppression, and increased CTLA-4 expression in tumor-infiltrating lymphocytes, which in experimental settings impair immune surveillance and foster invasive progression [104,105]. Increased breast cancer risk has been reported among rural women and farmers’ wives exposed to organophosphate insecticides through occupational activities, environmental exposure, or household use, especially in the case of chlorpyrifos and terbufos [13,100]. Furthermore, a pesticide-exposure dietary pattern dominated by chlorpyrifos, imazalil, malathion, and thiabendazole was positively associated with postmenopausal breast cancer, notably among overweight women [102]. Biomarker-based evidence shows that higher prediagnostic urinary levels of AMPA, the main glyphosate metabolite, predicted substantially increased breast cancer risk [101], while Brazilian case–control data indicate that chronic exposure to glyphosate, atrazine, and 2,4-D is linked to elevated breast cancer risk in crude analyses and to a markedly higher odds of lymph node metastasis, suggesting more aggressive disease [104]. Hospital-based work in Brazil further shows that chronically exposed breast cancer patients exhibit profound immune and nitrosative dysregulation, characterized by reduced Th1/Th17 cytokines, fewer tumor-infiltrating lymphocytes [99,103], increased CTLA-4, and altered NO/iNOS consistent with pesticide-induced immune compromise that may favor tumor progression [105]. Mechanistically, many implicated pesticides or metabolites act as endocrine disruptors (e.g., interfering with estrogen signaling, steroidogenesis, or AhR pathways), generate oxidative/nitrosative stress, and induce DNA damage or epigenetic alterations in mammary cells, all of which are biologically plausible routes to breast carcinogenesis and more aggressive phenotypes. At the same time, Table 1 deliberately incorporates several studies reporting null or non-significant associations to provide a balanced evidence base: a large prospective cohort of farmers’ wives in the agricultural health study found no significant overall association between occupational pesticide use and breast cancer [99], and a Brazilian case–control study found that adjusted odds ratios for chronic pesticide exposure were not statistically significant after covariate adjustment [103]. These null findings, alongside positive associations, underline major research gaps: limited power, crude exposure assessment, short follow-up for a long-latency cancer, poor characterization of mixtures and low-dose/non-monotonic effects, and sparse integration of repeated biomonitoring and mechanistic biomarkers.

### 3.3. Metabolic Disorders (Obesity, Diabetes, Metabolic Syndrome)

The epidemiological evidence summarized in Table 2 encompasses studies reporting both positive associations and null findings between pesticide exposure and major components of metabolic syndrome, and this balance is intentional to mitigate selective reporting; nonetheless, readers should note that publication bias toward positive results in the broader literature may still partially influence the overall evidence profile. Obesity, commonly defined by excess adiposity and indexed by body mass index (BMI), is a prevalent multifactorial condition associated with increased risk of cardiometabolic diseases and reduced life expectancy at the population level [110]. Epidemiological evidence consistently links pesticide exposure to increased adiposity across the course of life. Occupational exposure to atrazine has been associated with higher BMI among pesticide applicators [111]. Similarly, multiple cohorts demonstrated that prenatal exposure to DDT and its metabolites was associated with higher BMI in childhood and adulthood [18,20,21]. Cross-sectional studies further report positive associations between obesity or central obesity and organochlorine pesticides such as p,p’-DDE, o,p’-DDT, HCB, and chlorpyrifos in diverse adult populations [112,113,114,115]. Associations have also been observed for nonpersistent pesticides, particularly pyrethroids, with evidence of sex-specific effects [116]. During pregnancy, exposure to organochlorine pesticides has been consistently linked to excessive gestational weight gain, highlighting prenatal life as a sensitive window for obesogenic effects [117,118]. However, it is important to note that not all studies support these associations: a cross-sectional study in the United States found no significant association between urinary neonicotinoid metabolites including imidacloprid and clothianidin and obesity prevalence [119], and a cross-sectional study in Thailand found no significant association between chronic organophosphate pesticide exposure and insulin resistance as measured by HOMA-IR [120]; these null findings underscore that pesticide-metabolic associations are compound-specific and not universally generalizable across chemical classes. Mechanistically, pesticides with endocrine-disrupting properties may promote obesity by disrupting lipid metabolism, mitochondrial energy production, and neuroendocrine regulation of appetite and energy balance, thereby favoring fat accumulation [121,122]. Beyond adiposity, pesticide-related metabolic alterations frequently involve glucose regulation. Insulin resistance (IR) is a pathological state in which higher insulin levels are required to achieve normal metabolic responses and is commonly estimated in epidemiological studies using the HOMA-IR index [123]. IR represents a key mechanistic precursor to type 2 diabetes (T2D), a major global public health burden contributing to a broad spectrum of chronic health complications. Epidemiological studies consistently report positive associations between pesticide exposure and impaired glucose metabolism. Elevated serum concentrations of organochlorine pesticides, particularly p,p’-DDE, β-HCH, and HCB, have been associated with higher HOMA-IR, reduced insulin sensitivity, and increased odds of T2D across diverse populations [19,124,125,126,127]. Occupational exposure to multiple pesticide classes has also been linked to elevated diabetes risk among agricultural workers [128]. Moreover, environmental exposure to pyrethroids, assessed by urinary 3-PBA, showed a dose–response association with diabetes prevalence [17]. Pesticide-associated metabolic dysfunction may be driven by mitochondrial disruption, oxidative stress, and inflammation pathways, alongside impaired insulin signaling and pancreatic β-cell function, collectively fostering insulin resistance and the development of diabetic phenotypes [129,130].

Metabolic syndrome (MetS) is characterized by the co-occurrence of at least three out of five of the following clinical symptoms: obesity, hyperglycemia, raised triglycerides, decreased high-density lipoprotein (HDL) cholesterol, and hypertension [131]. Epidemiological studies consistently associate pesticide exposure with increased MetS risk. Urinary 2,5-dichlorophenol showed a dose–response relationship with MetS prevalence in U.S. adults [132]. Adipose and serum levels of organochlorine pesticides, including β-HCH, HCB, DDE, oxychlordane, trans-nonachlor, and Mirex, were significantly associated with higher odds of MetS or metabolically unhealthy phenotypes across adult populations in Spain and the United States [16,133,134,135].

**Table 2:** Epidemiological evidence linking pesticide exposure to metabolic disorders.

Study Design/Location	Sample Characteristics	Exposure Type/Biospecimen	Pesticide(s)	Key Findings	References
Obesity					
Cohort/ USA	8365 male pesticide applicators	Occupational exposure/self-reported (interview/ questionnaire)	Atrazine	Higher body mass index (BMI) observed among applicators with higher estimated lifetime atrazine exposure; suggests a possible link between triazine exposure and obesity	[111]
Cohort/ USA	218 women (18–40 years)	Pre-pregnancy exposure/Plasma	9 organochlorine pesticides	Total gestational weight gain was statistically associated with oxychlordane. The gestational weight gain curve was associated with p,p’-DDT. HCB was positively associated with total weight gain during pregnancy.	[136]
Cross-sectional/ Spain	429 adults (9.3% with T2D).	Environmental exposure/Serum	p,p’-DDE	p,p’-DDE level was significantly higher in people with BMI ≥ 25 kg/m^2^ than in people with normal BMI.	[112]
Cohort	240 children, 12 years	Prenatal exposure/Serum maternal collected during pregnancy	DDT DDE	Among boys, 10-fold increases in prenatal DDT and DDE concentrations were associated with increased BMI z-score (o,p’-DDT, adj-β = 0.37, 95% CI: 0.08, 0.65; p,p’-DDT, adj-β = 0.26, 95% CI: 0.03, 0.48; p,p’-DDE, adj-β = 0.31, 95% CI: 0.02, 0.59).	[20]
Cross-sectional/Sweden	988 adults	Environmental exposure/plasma	p,p’-DDE	p,p’-DDE levels were associated with increased fasting glucose, BMI, hypertension and left ventricular mass in separate models adjusted for sex.	[113]
Cross-sectional/ Iran	242 children/adolescents (6–18 years)	Environmental exposure/Urine	2,5-DCP	2,5-DCP: ↑ BMI z-score β = 0.07 (95% CI 0.04–0.10); ↑ waist circumference β = 0.79 (95% CI 0.54–1.03); ↑ obesity OR = 1.09 (95% CI 1.01–1.19).	[137]
Cross-sectional/ USA	Children (n = 784, 6–19 y) and adults (n = 1672, ≥20 y)	Environmental exposure/Urine	5 organophosphate esters	Exposure to select organophosphate esters differentially associated with increased or decreased general and central obesity in children and adults.	[138]
Prospective Cohort/ USA	n = 2334 pregnant women (8–13 wks)	Prenatal exposure/Plasma samples	11 organochlorine pesticides (OCPs)	OCPs such as DDT, DDE, HCB were associated with excessive weight gain during pregnancy.	[117]
Cohort/USA	511 middle-aged people	Prenatal exposure/serum	o,p’-DDT	Maternal o,p’-DDT associated with higher adult BMI: β = 0.59 kg/m^2^ per ln(ng/mL) (95% CI 0.17–1.00); waist circumference β = 1.19 cm (95% CI 0.26–2.13).	[18]
Cohort/ Spain	379 4–18 years	Prenatal exposure/Cord blood	HCB, p,p’-DDT, p,p’-DDE	HCB exposure in the third tertile, was associated with higher BMI (β = 0.24; 95% CI: 0.01, 0.47), waist-to-height ratio (WHtR) z-score (β = 0.27; 95% CI: 0.04, 0.51), and elevated body fat % (β per 10-fold increase = 4.21; 95% CI: 0.51, 7.92).	[21]
Cross-sectional/ India	100 adults	Environmental exposure/Adipose tissue	o,p’-DDT p,p’ DDD	o,p’-DDT (OR = 1.354 (1.022, 1.794)) and p,p’ DDD (OR = 1.070 (0.904–1.267) were strongly correlated with central obesity.	[114]
Cross-sectional/ USA	1675 adults > 19	Environmental exposure/Urine	Multiple Neonicotinoids/metabolites	No association between imidacloprid, clothianidin and N-desmethylacetamiprid and the occurrence of obesity. 5-Hydroxyimidacloprid was connected with an 11% increased incidence of overweight or obesity.	[119]
Cross-sectional/ South Korea	3692 adults	Environmental exposure/Urine	Pyrethroid metabolite (3-PBA)	3-PBA significantly associated with obesity (ORs = 1.0, 1.23, 1.43, 1.63) and increased BMI (ORs = 0.34, 0.46, 0.52).	[116]
Cross-sectional/ USA	7796 adults (4065 women) (≥20 years)	Environmental exposure/Urine	3-PBA	Among females, participants in the highest tertile of urinary 3-PBA had higher odds of obesity (OR = 1.22, 95% CI: 1.00, 1.48) compared to those in the lowest tertile after adjusting for covariates. Among males, the association was not statistically significant.	[17]
Cross-sectional/ Belgium & Luxembourg	Adults (n ≈ 989; 502 Belgium, 487 Luxembourg)	Environmental exposure/Hair	7 OCPs	Positive associations were found between obesity and HCB, chlorpyrifos, and β-HCH.	[115]
Cross-sectional/Finland	102 mother–child pairs	Environmental (Prenatal exposure)/Maternal plasma & cord plasma	p,p’-DDE	Pre-pregnancy BMI and weight change during pregnancy were positively associated with p,p’-DDE in children.	[118]
Insulin resistance/Diabetes					
Cohort/ Spain	107 women with a history of gestational diabetes mellitus	Environmental exposure/Serum	p,p’-DDE HCB	HCB, and p,p’-DDE were positively associated with HOMA-IR [(β = 0.40 (0.13, 0.67)], higher 2 h IRI, higher 2 h glucose, and lower insulin sensitivity.	[124]
Cross-sectional/ Canada	2172 Inuit adults	Environmental/ Serum	p,p’-DDE	p,p’-DDE was associated with increased risk of diabetes [OR = 2.5 (1.1, 6.0)].	[125]
Cross-sectional/ United Kingdom	192 adults. South Asians of Tamil or Telugu descent (n = 120) and European whites (n = 72)	Environmental exposure/ Plasma	p,p-DDE	South Asians had 9–30-fold higher p,p’-DDE levels than European whites. Diabetes strongly associated with elevated p,p’-DDE; OR = 7.00 (95% CI: 2.22–22.06)**.**	[139]
Case–control/ Thailand	866 cases with diabetes/1021 healthy controls (Farmers)	Occupational exposure/ Questionnaire	Multiple pesticides: insecticides, herbicides, fungicides, rodenticides	Diabetes risk was significantly increased with exposure to endosulfan (OR = 1.40), mevinphos (OR = 2.22), carbaryl (OR = 1.50), benlate (OR = 2.08), and rodenticides (OR = 1.35).	[128]
Case–control/USA	793 middle-aged women	Environmental exposure/Plasma	3 OCPs HCB, β-HCH p,p’-DDE	High vs. low tertile pesticide exposure increased type 2 diabetes risk: HCB OR = 1.67 (1.24, 2.23; Ptrend < 0.001), β-HCH OR = 3.62 (2.57–5.11; Ptrend < 0.001), and p,p’-DDE OR = 1.55 (1.13–2.13; Ptrend < 0.001).	[126]
Cross-sectional/ USA	2796 adults aged 20–79 years	Environmental exposure/Urine	3-PBA	Significant dose–response association between urinary 3-PBA levels and diabetes prevalence. Highest vs. lowest quartile: OR = 2.18 (95% CI: 1.18–4.03)**.**	[17]
Case–control/ Germany	132/263 adults, older than 45 years	Environmental exposure/Serum	HCB, p,p’-DDE β-HCH	HCB OR = 1.42 (1.11; 1.82), 4,4′-DDE (OR = 1.22 (1.00; 1.48) were significantly (*p* < 0.05) associated with an increased odds of having incident diabetes.	[127]
Cross-sectional/ Pakistan & Cameroon	904 adults: 592 exposed (lived in OP-sprayed agricultural area) & 312 unexposed	Occupational exposure/Plasma	Malathion, chlorpyrifos, parathion	In both population samples, pesticide exposure was associated with marked pancreatic and metabolic dysregulation, including significantly elevated fasting glucose, insulin, and HOMA-IR.	[140]
Cross-sectional/ Thailand	36 sprayers and 42 nonsprayers	Occupational exposure/Urine	Mix organophosphates	No significant association showed between chronic organophosphates exposure and HOMA-IR.	[120]
Case–control/ Algeria	361 adults (180 cases with T2D and 181 non-diabetic)	Environmental exposure/Plasma	p,p’-DDE; HCB	Exposure to p,p’-DDE OR = 12.58 (95% CI: 4.76–33.26) and HCB OR = 3.69 (1.90–7.15) was associated with an increased risk of type 2 diabetes.	[141]
Case–control/ India	100 normal/100 prediabetic/100 new diabetics	Environmental exposure/ Whole blood	β-HCH Dieldrin p,p’-DDE	Insulin resistance positively correlated with β-HCH and dieldrin. Adjusted ORs for diabetes risk: β-HCH (OR = 2.70)**,** dieldrin (OR = 2.83), p,p’-DDE (OR = 2.55).	[114]
Cross-sectional/ Canada	419 women	Environmental exposure/ Serum	p,p’-DDT p,p’-DDE	the highest detectable levels of DDT (PR = 1.93, 1.17, 3.19), and tertiles of DDE (PR = 3.58, 1.10, 11.70) were significantly associated with prevalent T2DM in the fully adjusted model.	[142]
Case–control/ USA	442 youth 10–22 years	Environmental exposure/Plasma	p,p’-DDE, p,p’-DDT, hexachlorobenzene, tNONA	p,p’-DDE and tNONA were associated with higher odds of type 1 diabetes with normal insulin sensitivity (ORs ≈ 2.0–2.5 for 2nd/3rd tertiles).	[143]
Metabolic syndrome					
Cross-sectional/USA	1706 non-diabetic adults (20–79 years)	Environmental exposure/Urine	2,5-DCP	Dose-dependent increase in MetS prevalence by urinary 2,5-DCP quartile; Adjusted ORs: Q3 1.47 (95% CI 1.02–2.14); Q4 1.56 (95% CI 1.10–2.23) vs. Q1.	[132]
Combined cross-sectional + 10 yr longitudinal/Spain	n = 387 adipose baseline n = 154 longitudinal	Environmental exposure/Adipose tissue	β-HCH, HCB	After adjusting for confounders, β-HCH and HCB were independently associated with an increased risk of being metabolically compromised [HRs =1.28, 95% CI =1.01–1.61 (β-HCH); 1.26, 95% CI =1.00–1.59 (HCB)].	[133]
Cross-sectional/USA	548 adults (no diabetes)	Environmental exposure/Serum	9 OCPs	Several OCPs were found to have significant associations with metabolic syndrome. Oxychlordane [OR =2.09 (1.07–4.07)]; tNONA [3.19 (1.45–7.00)]; HCB [OR =6.15 (1.66–22.88)].	[134]
Cross-sectional/ Spain	1374 adults	Environmental exposure/Serum	HCB β-HCH	Higher serum HCB [PRs up to 2.1 (95% CI 1.0–4.3)], β-HCH [PRs up to 2.8 (95% CI 1.1–6.7)] were strongly associated with an increased prevalence of metabolically unhealthy phenotype among normal-weight adults.	[16]
Cross-sectional/USA	601 adults (18–84 y)	Environmental exposure/Serum	OCPs (HCB, DDE, and Mirex)	HCB (tertile3: β = 0.59) and DDE (tertile3: β = 1.19) strongly associated with risk of the MetS.	[135]

OCPs: organochlorine pesticides; DDT: dichlorodiphenyltrichloroethane; DDE: dichlorodiphenyldichloroethylene; HCB: Hexachlorobenzene; 2,5-DCP: 2,5-dichlorophenol; 3-PBA: 3-phenoxybenzoic acid; β-HCH: β-hexachlorocyclohexane; tNONA: trans-nonachlor; T2D: type 2 diabetes; MetS: Metabolic syndrome; ↑ increased.

### 3.4. Thyroid Disorders

The thyroid gland plays a key role in controlling metabolism, growth, thermogenesis, and neurodevelopment through tightly controlled thyroid hormone homeostasis maintained by the hypothalamic–pituitary–thyroid (HPT) axis [144]. Thyroid disorders are among the most common endocrine diseases in the world. They affect around 10% of the population and are a major cause of multiple complications, with especially severe consequences during pregnancy, childhood, and aging [145]. Table S3 presents epidemiological evidence linking pesticide exposure to thyroid toxic effects throughout the life-course. Multiple cohort and case–control studies reported increased risks of thyroid cancer, especially papillary thyroid carcinoma, associated with exposure to several pesticides, including atrazine, malathion, metalaxyl, and lindane, with stronger effects observed in women [24,146,147,148]. Beyond thyroid cancer, several cross-sectional and longitudinal studies have documented changes in circulating thyroid hormones caused by pesticides. In adults, pregnant women, and neonates, elevated serum or cord blood concentrations of OCPs and their metabolites, such as p,p’-DDE, β-HCH, aldrin, dieldrin, and nonachlors, have been linked to changes in TSH, free T4, total T4, and total T3 [149,150,151,152]. Furthermore, population-based studies revealed that living in regions with high pesticide use was linked to higher rates of hypothyroidism, thyrotoxicosis, thyroiditis, and goiter [153].

**Figure 3:**
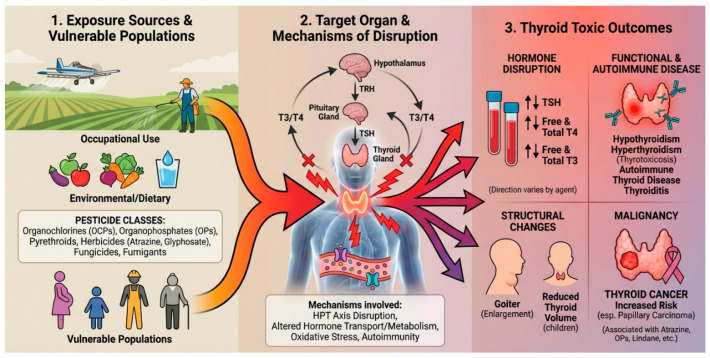
Thyroid disruption and toxic outcomes associated with pesticide exposure.

Non-persistent pesticides, such as organophosphates and pyrethroids, have also been linked to subclinical thyroid dysfunction and decreased thyroid volume, a structural alteration observed not only in children but also in adult populations (Figure 3). This suggests that thyroid development and function may be affected even at low environmental exposure levels across the lifespan [154,155,156]. Pesticides can impair thyroid function and cause long-term thyroid disorders by interfering with hormone transport proteins, receptor binding, hepatic metabolism of T3 and T4, and deiodinase activity [157,158]. Furthermore, oxidative stress, immunotoxicity, and epigenetic modifications have all been connected to thyroid cancer [159,160].

### 3.5. Neurodevelopmental Problems

#### 3.5.1. Attention Deficit Hyperactivity Disorder (ADHD)

Epidemiological research suggests a link between prenatal and postnatal exposure to pesticides, specifically pyrethroids and organophosphates, and ADHD, though findings are inconsistent (Table 3). High pyrethroid exposure, typically assessed via urinary 3-PBA, has been associated with increased ADHD traits in young children and school-aged children [161,162], but some follow-up work indicates that these associations may fade or become harder to detect by age 5 [163], and burden-of-disease modeling estimates that roughly 7–18% of European ADHD cases could be attributable to pyrethroid exposure [164]; for organophosphates, results are mixed, with a large Norwegian cohort finding no association between prenatal dialkylphosphate metabolites and ADHD [165], whereas more highly exposed groups, such as Egyptian adolescent chlorpyrifos applicators and rural Chinese children, show strong positive associations between chlorpyrifos biomarkers and ADHD symptoms [23,25]. These epidemiological patterns are biologically plausible given that pyrethroids alter voltage-gated sodium channels and disrupt dopaminergic signaling, promoting hyperactivity and impulsivity, while organophosphates inhibit cholinesterase and perturb GABAergic, glutamatergic, and dopaminergic neurotransmission and induce oxidative stress, all of which can impair attention and executive control.

#### 3.5.2. Autism Spectrum Disorder (ASD)

Epidemiological evidence increasingly links pesticide exposure, especially during prenatal and early-life windows, to ASD (Table 3). Large population-based studies using agricultural application data report modest but consistent elevations in ASD risk with residential proximity to intensive pesticide use, including glyphosate, organophosphates (chlorpyrifos, diazinon, malathion), pyrethroids (permethrin, bifenthrin), avermectins, methyl bromide, and myclobutanil, with stronger associations for ASD with comorbid intellectual disability and for first year of life exposure [22,171,172]. Prospective biomarker studies add compound-specific support: early-pregnancy urinary chlorpyrifos/oxon and possibly diazinon predicted more autistic traits at 11 years [170]; in a high-risk cohort, higher prenatal dimethylthiophosphate was suggestively associated with ASD in girls only [168], and pyrethroid metabolite 3-PBA showed at most a small, imprecise ASD risk increase [169]; elevated maternal p,p’-DDE, but not PCBs, increased ASD risk in Finland, particularly ASD with intellectual disability [167]. These associations are mechanistically plausible because many implicated pesticides inhibit acetylcholinesterase, induce oxidative stress, disrupt monoamine and glutamate signaling, alter microglial activation and mitochondrial function, act as endocrine disruptors, and perturb gut microbiota and barrier integrity pathways relevant to ASD neurodevelopment [22,167,168,170,171,172].

## 4. Future Directions and Research Gaps

Despite substantial progress in understanding EDPs, important scientific and methodological gaps continue to limit causal interpretation, cross-study comparability, and translation into public health policy. Advancing this field will require a shift from largely associative and single-chemical approaches toward integrative, life-course, and mechanism-informed research frameworks that better reflect real-world exposure conditions.

First, improved exposure assessment must be treated as a central priority. A major limitation of the current literature is the frequent reliance on single time-point biomarker measurements, self-reported pesticide use, or residential proximity indicators. These methods often fail to capture temporal variability, cumulative burden, and exposure during biologically sensitive windows, particularly for non-persistent pesticides with short half-lives. Future studies should prioritize repeated biomonitoring across key life stages, including preconception, pregnancy, infancy, childhood, puberty, and adulthood. Whenever possible, biomarker data should be integrated with dietary information, occupational histories, household use patterns, environmental monitoring, and geospatial modeling to reconstruct more accurate individual exposure trajectories.

Second, mixture effects require much greater emphasis. In real-world settings, humans are not exposed to one pesticide at a time, but rather to complex combinations of persistent and non-persistent pesticides, often alongside other endocrine-disrupting chemicals. Yet much of the epidemiological literature remains dominated by single-compound analyses. This reductionist approach is unlikely to capture additive, synergistic, antagonistic, or non-monotonic effects that may be central to endocrine disruption. Experimental toxicology demonstrates that pesticide cocktails frequently display complex interaction dynamics; for instance, multi-component pesticide combinations can produce concentration-additive or antagonistic effects on thyroid receptor signaling and AhR transactivation, where specific fungicides (e.g., bitertanol) exert masking or inhibitory actions that modulate overall mixture activity [34]. Furthermore, evidence from mixture modeling studies enables a direct comparison between individual and cumulative impacts: advanced analytical frameworks such as Weighted Quantile Sum (WQS) regression, Bayesian Kernel Machine Regression (BKMR), and quantile g-computation demonstrate that joint exposure to pesticide mixtures yields stronger cumulative associations with obesity, metabolic dysfunction, and postmenopausal breast cancer than single-compound models alone, while successfully identifying primary driving chemicals within complex real-world exposures [102,116]. Future studies should therefore apply advanced mixture methods. In particular, it will be important to identify combinations of pesticides that converge on shared endocrine targets, including estrogenic, anti-androgenic, thyroid-disrupting, or obesogenic pathways.

Third, stronger integration of mechanistic biomarkers into human studies is essential for improving causal inference. One of the major challenges in the EDP field is the gap between exposure metrics and overt disease outcomes, which often emerge after long latency periods and are influenced by multiple confounders. To address this, future epidemiological studies should incorporate biomarkers that reflect early biological effect and pathway perturbation, including sex steroid and thyroid hormone panels, inflammatory mediators, oxidative stress markers such as 8-OHdG and lipid peroxidation products, mitochondrial dysfunction indicators, and receptor-related signaling markers. Epigenetic endpoints, including DNA methylation, histone modifications, and non-coding RNAs, should also be incorporated wherever feasible, particularly in birth cohorts and reproductive studies. The integration of transcriptomics, metabolomics, proteomics, and epigenomics may help identify early signatures of susceptibility and clarify how shared mechanisms connect EDP exposure to diverse disease phenotypes.

Fourth, future research should adopt a more explicit life-course perspective. Endocrine systems are especially vulnerable during fetal development, infancy, childhood, adolescence, pregnancy, and reproductive aging. To establish a balanced life-cycle risk gradient, future cohorts must evaluate how EDP exposures interact with distinct endocrine windows, including pubertal hypothalamic–pituitary–gonadal maturation and perimenopausal/postmenopausal ovarian and thyroid senescence. However, many existing studies remain cross-sectional or focus on adult disease without adequately accounting for developmental origins. Longitudinal cohort studies with repeated exposure and outcome measurements are especially needed to determine whether early-life pesticide exposure contributes to later reproductive dysfunction, obesity, diabetes, thyroid disease, or endocrine-related cancers. Such designs would also help clarify issues of latency, persistence, reversibility, and developmental programming.

Fifth, sex-specific and sex-dependent analyses should become standard practice. Evidence increasingly suggests that males and females may differ not only in exposure patterns, but also in toxicokinetics, endocrine physiology, target organ susceptibility, and downstream disease manifestation. Nonetheless, many studies still combine sexes or treat sex only as a covariate rather than a biologically meaningful modifier. Future work should be designed and powered to evaluate sex-stratified associations, sex-by-exposure interactions, and potentially distinct mechanisms in male and female populations. This is particularly important for reproductive outcomes, thyroid disorders, adiposity, and neurodevelopmental endpoints, where endocrine physiology differs substantially by sex and developmental stage.

Sixth, transgenerational and intergenerational effects remain a key frontier in EDP research. Experimental evidence suggests that some pesticides may induce persistent epigenetic alterations that extend beyond directly exposed individuals, yet human evidence remains sparse. Multigenerational cohort studies, family-based designs, and analyses leveraging archived maternal biospecimens could help determine whether pesticide-induced endocrine disruption influences health across generations. Distinguishing true transgenerational inheritance from intergenerational exposure effects will be essential in this context, but both have important implications for risk assessment and prevention.

Seventh, greater harmonization of outcome definitions and exposure metrics is needed to improve comparability across studies. The current literature is characterized by substantial heterogeneity in biomonitoring methods, exposure classification, diagnostic criteria, and clinical endpoint definitions. This heterogeneity complicates meta-analysis and weakens evidence synthesis. Standardized protocols are needed for evaluating reproductive dysfunction, thyroid abnormalities, metabolic syndrome, and neurodevelopmental outcomes such as ADHD and ASD. For cancer outcomes, greater consistency in distinguishing aggressive from indolent disease would also strengthen interpretation. Similarly, validated biomarker panels, standardized reporting units, and harmonized reference intervals for pesticide metabolites would improve comparability across cohorts and regions.

Eighth, more attention should be directed toward vulnerable and underserved populations, with explicit stratification between occupational and non-occupational cohorts. High-dose occupational exposures among agricultural applicators often yield distinct linear dose–response patterns for high-grade outcomes [94,111], whereas general populations subjected to low-dose chronic dietary residues frequently exhibit non-monotonic dose–response relationships or subtle metabolic dysfunction [102,126]. Consequently, future research must incorporate systematic population stratification, across exposure pathways (occupational vs. dietary), age, sex, socioeconomic status, and dietary patterns (e.g., conventional vs. organic food intake), to accurately characterize dose–effect gradients and resolve conflicting epidemiological evidence. Agricultural workers, pesticide applicators, women involved in pesticide mixing or laundering contaminated clothing, rural residents, and low-income communities often experience higher and more sustained exposure burdens, yet they remain underrepresented in biomonitoring-intensive and longitudinal research. Environmental justice considerations should therefore be more fully incorporated into future study design. Research should also examine how pesticide exposure interacts with nutrition, psychosocial stress, access to healthcare, and co-exposures to other environmental contaminants, especially in populations already facing structural health inequities.

## 5. Conclusions

The evidence reviewed demonstrates that EDPs are associated with substantial risks to human health through complex, interrelated mechanisms that disrupt hormonal signaling at multiple levels. The convergence of epidemiological findings across diverse populations and study designs, supported by biologically plausible mechanistic pathways, strengthens the case for possible or probable causal relationships for some outcomes, although causality remains uncertain for others. However, a balanced perspective requires acknowledging key limitations within the available literature, including potential publication bias favoring positive associations, observed inconsistencies in epidemiological evidence across certain disease endpoints, and challenges in directly extrapolating high-dose animal and in vitro models to real-world, low-dose human exposures. Importantly, the present review extends beyond prior single-outcome or single-class summaries by integrating mechanistic and epidemiological evidence across reproductive, oncologic, metabolic, thyroid, and neurodevelopmental domains within a unified endocrine disruption framework. Particularly concerning are effects during critical developmental windows, including prenatal and early childhood periods, where exposure may produce irreversible consequences with transgenerational implications through epigenetic mechanisms. Ultimately, a unified toxicological framework emerges wherein molecular initiating events—specifically nuclear receptor dysregulation (ER, AR, TR, PPAR, AhR), non-genomic receptor interference, and steroidogenic enzyme inhibition—converge on systemic key events including mitochondrial impairment, persistent reactive oxygen species generation, chronic inflammation, and epigenetic reprogramming (DNA methylation, histone modification, altered microRNA profiles). Rather than acting through isolated pathways, these interconnected mechanisms establish a shared pathophysiological foundation through which chronic, low-dose exposure to pesticide mixtures disrupts developmental programming and cellular homeostasis, driving the continuum of adverse health outcomes observed across reproductive, oncologic, metabolic, thyroid, and neurodevelopmental domains. To advance this field, future research must prioritize large prospective cohort studies with repeated biomonitoring across life stages, improved characterization of pesticide mixtures and cumulative exposures using AI-driven analytical approaches, the integration of mechanistic biomarkers derived from multi-omics and 3D organoid models, and sex-specific and life-course analytical approaches. Standardized exposure assessment methods and harmonized outcome definitions are essential for synthesizing evidence across studies. Taken together, this integrative perspective underscores that EDP-related health effects should not be viewed as isolated endpoint-specific phenomena, but rather as interconnected manifestations of shared endocrine and developmental perturbation. Given the ubiquitous nature of pesticide exposure and the fundamental role of endocrine systems in human health, these findings underscore the urgent need for precautionary regulatory approaches, reduced agricultural dependence on endocrine-disrupting compounds, and targeted interventions to protect susceptible populations, particularly pregnant women, children, and occupationally exposed workers. More broadly, the framework proposed here may help guide future interdisciplinary research and support more mechanism-informed, health-protective regulatory policy.

## Figures and Tables

**Table 1:** Epidemiological studies examining the association between pesticide exposure and cancer.

Study Design/Location	Sample Characteristics	Exposure Type/Biospecimen	Pesticide(s)	Key Findings	References
Testicular cancer					
Registry-based case–control/Denmark, Finland, Norway, Sweden	9569 testicular germ cell tumor (TGCT) cases/32,028 controls	Parental occupational exposure/History from census or pension data linked to a Job-Exposure Matrix (FINJEM).	General occupational pesticide exposure (fungicides, herbicides, insecticides).	No overall association found between maternal or paternal occupational pesticide exposure and TGCT risk in sons.	[89]
Case–control/France	304 TGCT cases/274 controls.	Self-reported domestic use/Questionnaire data collected from mothers/relatives	Domestic insecticides, fungicides, and herbicides	Statistically significant increased risk of TGCT overall (OR = 1.73) and Non-Seminoma subtype (OR = 2.44) associated with domestic use of fungicides.	[92]
Multicenter case–control/(TESTIS study)/France	454 TGCT cases/670 controls	Occupational exposure/Self-reported	Occupational Agricultural	Increased risk of TGCT observed in agricultural and animal husbandry workers.	[14]
Nationwide Case–control (TESTIS study)/France	454 TGCT cases/670 controls	Parental occupational exposure/Self-reported	General occupational exposure inferred from job titles (e.g., farmers, crops).	Pesticides: “Specialized farmers” and “Crops” (sensitivity analysis) job titles associated with increased risk, suggesting a link to pesticide exposure.	[15]
Nested case–control/Denmark	332 mother-son pairs (65 TGCT cases and 267 matched controls)	Parental exposure/Maternal serum samples collected during pregnancy (biobanked).	Organochlorine pesticides (OCPs) and polychlorinated biphenyls (PCBs).	Prenatal exposure to OCPs and PCBs was not associated with the risk of developing TGCT later in life.	[90]
Prostate cancer					
Case–control; British Columbia, Canada	1153 prostate cancer cases/3999 Control	Occupational exposure/job exposure matrix	Multiple (180) active ingredients (e.g., DDT, lindane, simazine, 2,4-D, 2,4-DB, others)	Higher cumulative exposure to several pesticides (notably DDT, lindane, simazine, 2,4-D, 2,4-DB, dinoseb amine, carbaryl, malathion, endosulfan) was associated with increased prostate cancer risk, generally with dose–response patterns.	[93]
Prospective cohort/USA	54,412 male pesticide applicators; 1962 incident prostate cancers, including 919 aggressive cases	Self-reported lifetime pesticide use at enrollment and 5-year follow-up	48 specific pesticides; key: organophosphate insecticides fonofos, malathion, terbufos; organochlorine insecticide aldrin	No clear association with total prostate cancer overall. Aggressive prostate cancer risk was increased in the highest exposure quartile for fonofos (RR 1.63), malathion (RR 1.43), terbufos (RR 1.29), and aldrin (RR 1.49). For fonofos and aldrin, risks were stronger among men with a family history of prostate cancer.	[94]
Prospective cohort/USA	883 aggressive prostate cancer cases	Occupational exposure/Self-reported pesticide use (questionnaires)	Dimethoate (organophosphate insecticide), triclopyr (herbicide) among 39 pesticides	Dimethoate use was positively associated with aggressive prostate cancer (HR ~1.4); triclopyr use was inversely associated (HR ~0.7), strongest for ≥4 years use; other pesticides showed no clear associations.	[95]
Cross-sectional/ Seoul, Korea	1305 men ≥ 40 years without prostate cancer	Environmental exposure/Urine & serum	3-PBA (pyrethroid metabolite)	Highest urinary 3-PBA associated with ~2-fold higher odds of elevated total prostate-specific antigen (≥4 ng/mL) and low prostate-specific antigen ratio (<15%), especially in men with normal kidney function, suggesting possible pyrethroid-related prostate damage.	[96]
Case–control/France mainland and French West Indies	160 men with prostate cancer	Environmental exposure/Periprostatic adipose tissue	29 OCPs	No positive association between any pesticide and aggressive prostate cancer; mirex was inversely associated with aggressiveness.	[97]
Prospective cohort/USA	52,625 pesticide applicators	Occupational exposure/Self-reported carbaryl use (questionnaires)	Carbaryl (carbamate insecticide)	Total prostate cancer: no clear association. Aggressive prostate cancer: no association unlagged, but with 30-year lag, highest exposure linked to higher risk (RR 1.56, 95% CI 1.18–2.08).	[98]
Breast cancer					
Prospective cohort/USA	47,640 women; 1966 incident breast cancer cases	Occupational exposure/Self-reported (Questionnaires)	Multiple pesticides	Occupational pesticide use (~2% ever exposed) was not significantly associated with breast cancer (total, invasive, in situ, or by hormone receptor status).	[99]
Cohort/USA	30,594 farmers’ wives without breast cancer; 1081 incident invasive breast cancers.	Occupational exposure/Self-reported (Questionnaires)	Multiple insecticides	Wives’ overall insecticide use was not associated with breast cancer, but ever-use of chlorpyrifos (HR 1.4, 95% CI 1.0–2.0) and terbufos (HR 1.5, 95% CI 1.0–2.1) was linked to higher risk, especially for premenopausal breast cancer.	[13]
Case–control/USA	155 postmenopausal breast cancer cases/150 controls	Histories linked to commercial pesticide reports and land use data.	Multiple OCPs and Ops	No association between breast cancer and OCPs. Ambient exposure to chlorpyrifos was linked to ~3-fold higher breast cancer odds vs. unexposed (adjusted OR 3.22; 95% CI 1.38–7.53), robust to latency exclusions.	[100]
Multiethnic Cohort/USA	124 breast cancer cases/126 matched controls	Environmental exposure/Urine	AMPA, main metabolite of glyphosate	Women in the highest vs. lowest quintile of AMPA excretion had OR 4.49 (95% CI 1.46–13.77; p-trend = 0.029), suggesting higher AMPA exposure is associated with increased breast cancer risk.	[101]
Prospective cohort (NutriNet-Santé)/France	13,149 postmenopausal women; 169 incident breast cancer cases	Modeled dietary exposure to 25 pesticide active substances from FFQ (conventional vs. organic foods) combined with residue database.	Mixtures of fungicides, insecticides, herbicides; key pattern 1 driven by chlorpyrifos, imazalil, malathion, thiabendazole; pattern 3 = low synthetic pesticide exposure/higher spinosad.	A low-synthetic-pesticide profile (Component 3) was associated with lower postmenopausal breast cancer risk (Q5 vs. Q1 HR 0.57; 95% CI 0.34–0.93). A high-exposure profile to chlorpyrifos/imazalil/malathion/thiabendazole (Component 1) was linked to increased risk only in overweight/obese women (BMI ≥ 25 kg/m^2^; Q5 vs. Q1 HR 4.13; 95% CI 1.50–11.44).	[102]
Case–control study/Brazil	191 breast cancer cases/185 controls	Environmental and occupational exposure/Questionnaire	Mixed/unspecified pesticides	Women reporting pesticide use for >10 years had higher odds of breast cancer than those never/≤10 years exposed, but this was not statistically significant after adjustment (adjusted OR 1.40; 95% CI 0.85–2.49).	[103]
Case–control/Brazil	728 women for risk analysis.	Occupational exposure/Urine& Questionnaires	Mainly herbicides glyphosate, atrazine, and 2,4-D.	Exposed women had higher crude breast cancer risk (OR 1.58; 95% CI 1.18–2.13), attenuated and non-significant after adjustment (OR 1.30; 95% CI 0.87–1.95). In the substudy, 53% of urine samples were pesticide-positive, including women only handling dilution and washing personal protection equipment/clothes.	[104]
Hospital-based observational study/Brazil	215 women with breast cancer (128 occupationally exposed rural; 87 unexposed urban)	Occupational exposure/plasma, tumor tissue, and pooled urine	Mainly herbicides; region dominated by glyphosate	Exposed women showed altered plasma proteome and marked immune/nitrosative dysregulation: lower systemic IL-1β, IL-12, TNF-α, IL-17A; reduced IL-12 and TNF-α in tumors; fewer TILs; higher CTLA-4 in TILs; lower NOx and iNOS, indicating pesticide-related immune compromise in breast cancer.	[105]

OCPs: Organochlorine pesticides; 3-PBA: 3-phenoxybenzoic acid; AMPA: Aminomethylphosphonic acid; OR: odds ratio; HR: hazard ratio; RR: relative risk; CI: confidence interval; BMI: body mass index; FFQ: food frequency questionnaire; TILs: tumor-infiltrating lymphocytes; CTLA-4: cytotoxic T-lymphocyte-associated protein 4; iNOS: inducible nitric oxide synthase; NOx: nitrite/nitrate.

**Table 3:** Epidemiological studies examining the association between pesticide exposure and ADHD and autism.

Study Design/Location	Sample Characteristics	Exposure Type/Biospecimen	Pesticide(s)	Key Findings	References
ADHD					
Prospective cohort/Denmark	948 mother–child pairs	Prenatal exposure/Maternal urine	Chlorpyrifos metabolite (TCPY) & pyrethroid metabolites (via 3-PBA, trans-DCCA)	Maternal 3-PBA and trans-DCCA were associated with higher ADHD risk, particularly when 3-PBA co-occurred with TCPY.	[161]
Cohort/Egypt	64 adolescents: 39 pesticide applicators & 25 non-applicators	Occupational exposure/urine	Chlorpyrifos metabolite (TCPY)	Applicators showed higher TCPY levels, greater cholinesterase inhibition, and more ADHD symptoms than non-applicators, with a clear dose–response relationship.	[23]
Nested case–control within national birth cohort/Finland	359 ADHD cases, 359 controls	Prenatal exposure/Maternal serum.	p,p’-DDE (DDT metabolite; organochlorine insecticide).	No association between maternal DDE (75th/90th percentile or continuous) and offspring ADHD diagnosis.	[166]
Prospective cohort/South Korea	524 mother–child pairs	Prenatal exposure/Urine	Multiple pyrethroid insecticide via 3-PBA	Doubling of prenatal and age-2 3-PBA was associated with higher ADHD symptom scores at age 6; doubling at ages 4 and 6 was associated with higher ADHD scores at age 8.	[162]
Cohort/Norway	259 preschool ADHD cases and 547 reference children	Prenatal exposure/Maternal urine	Organophosphorus pesticides: (DAP) metabolites summed as ∑DEP and ∑DMP	No association between prenatal ∑DEP or ∑DMP and preschool ADHD; quartile analyses near null, slightly inverse, non-monotonic.	[165]
Cross-sectional/China	673 children aged 1–6 years	Environmental exposure/Urine	Chlorpyrifos	21.4% had detectable urinary chlorpyrifos. Higher chlorpyrifos exposure was associated with higher ADHD risk. Vitamin D was protective and partially mediated the chlorpyrifos–ADHD association (~19% mediation).	[25]
Prospective cohort/Denmark	614 pregnant women 814 children at 5 years	Prenatal exposure/Urine	Chlorpyrifos via TCPY; multiple pyrethroids via 3-PBA/trans-DCCA.	No statistically significant associations between prenatal or child TCPY/3-PBA (alone or combined) and ADHD score ≥ 90th percentile at age 5.	[163]
Autism					
Nested case–control within national birth cohort/Finland	778 childhood autism cases/778 controls	Prenatal exposure/Serum	Organochlorine insecticide metabolite p,p’-DDE (from DDT)	Maternal p,p’-DDE > 75th percentile associated with higher odds of autism (OR 1.32, 95% CI 1.02–1.71), and >2-fold higher odds for autism with intellectual disability (OR 2.21, 95% CI 1.32–3.69).	[167]
Prospective high-risk cohort/USA	203 mother–child pairs at elevated familial ASD risk;	Prenatal exposure/Urine	Organophosphate pesticides (OPs)	No association between prenatal OP metabolites and ASD or other developmental concerns when sexes combined. In girls only, higher DMTP showed a suggestive increase in ASD risk (OR per doubling 1.64; 95% CI 0.95–2.82), not seen in boys.	[168]
Case–control/USA	2961 cases/35,370 controls	Prenatal exposure/Pesticide use reporting	11 high-use pesticides	Prenatal exposure to several pesticides was associated with modestly higher ASD odds (e.g., glyphosate OR 1.16; chlorpyrifos 1.13; diazinon 1.11; permethrin 1.10). For ASD with intellectual disability, prenatal and especially first-year exposure showed larger increases in risk (e.g., first-year glyphosate OR 1.60).	[22]
Prospective cohort/USA	201 mother–child pairs at elevated familial ASD risk	Prenatal exposure/Urine	Pyrethroid metabolite (3-PBA)	A higher level of 2nd-trimester 3-PBA was associated with a relative risk ratio (RRR) of approximately 1.5 for ASD (95% CI 0.89–2.51).	[169]
Prospective birth cohort/France	185 mother–child pairs	Prenatal exposure/Urine	Organophosphate insecticides and their metabolites	No association for DAPs, terbufos, or metabolites. Detection of chlorpyrifos or chlorpyrifos-oxon in maternal urine was associated with higher CAST scores (IRR 1.27; 95% CI 1.05–1.52), stronger in boys (IRR 1.39; 95% CI 1.07–1.82); high diazinon showed a similar, weaker pattern.	[170]
Population-based case–control/Spain	52,393 residents; 2821 ASD cases	Environmental exposure: districts classified as high vs. low pesticide use	Mixed agricultural pesticides	ASD prevalence was higher in high- vs. low-use areas (1.03 vs. 0.76 per 100; OR 1.34, 95% CI 1.24–1.44). Adjusted logistic regression: living in high-use areas OR 1.52 (95% CI 1.41–1.64); males had a higher risk than females (OR 2.41, 95% CI 2.21–2.62).	[171]

TCPY: 3,5,6-trichloro-2-pyridinol; 3-PBA: 3-phenoxybenzoic acid; trans-DCCA: trans-3-(2,2-dichlorovinyl)-2,2-dimethylcyclopropane-1-carboxylic acid; DAP: Dialkylphosphate; ∑DEP: Total diethylphosphate; ∑DMP: Total dimethylphosphate; DMTP: dimethylthiophosphate.

## Data Availability

Data are included within the text and Supplementary Materials [173,174,175,176,177,178,179,180,181,182,183,184,185,186,187,188,189,190,191,192].

## Supplementary Materials

The following supporting information can be downloaded at: https://www.mdpi.com/article/10.3390/ijms27156928/s1.
