# Supplementary material for: Endocrine-Disrupting Pesticides as Drivers of Human Disease: Mechanistic Toxicology and Life-Course Health Effects
Source: Int J Mol Sci. 2026 Aug 1;27(15):6928. doi: 10.3390/ijms27156928 (PMC13466750; doi:10.3390/ijms27156928)
Supplement: Supplementary file 1 [file ijms-27-06928-s001.zip › ijms-4454568-supplementary.pdf]

Review

Endocrine-Disrupting Pesticides as Drivers of Human Disease: Mechanistic Toxicology and Life-Course Health Effects

Nour El-Hoda Zidan [1], Tarek Alshaal [2,*], Nevien Elhawat [2,*], Osama Elhamalawy [3], Farag Malhat [4] and Fawzy Eissa [3]

[1] Pesticides Chemistry and Toxicology Department, Faculty of Agriculture, Kafrelsheikh University, Kafr El-Sheikh 33516, Egypt; nourelhoda_az@hotmail.com
[2] Department of Food Biotechnology, Albert Kázmér Mosonmagyaróvár Faculty, Széchenyi István University, 9026 Gy˝or, Hungary
[3] Environment and Bio-agriculture Department, Faculty of Agriculture, Al-Azhar University, Cairo 11884, Egypt; osamaelhamalawy@yahoo.com (O.E.); fawzy.eissa@yahoo.com (F.E.)
[4] Central Agricultural Pesticide Laboratory, Pesticide Residues and Environmental Pollution Department, Agricultural Research Center, Giza 12618, Egypt; farag_malhat@yahoo.com
* Correspondence: alshaal.tarek.ali.ahmed.ibrahim@sze.hu (T.A.); elhawat.nevien.adel.ismaeil@ga.sze.hu (N.E.)

Supplementary Data

Table S1: Mechanisms of Action of Endocrine-Disrupting Pesticides, Affected Hormonal Pathways, and Biological Effects.

Primary Mechanism	Specific Action / Target	Representative Pesticides	Biological Effects & Affected Pathways	References
**Nuclear Receptor Modulation**	Estrogen receptor (ER) Interaction	DDT, Methoxychlor, Glyphosate, Linuron, Cyfluthrin	Binds to and modulates estrogen receptors (ESR1, ESR2). Actions can be agonistic (estrogenic) or antagonistic (anti-estrogenic), leading to altered gene expression (e.g., *Pgr*, *Ccnd1*, *pS2*), proliferation of hormone-sensitive cells (e.g., MCF-7), and potential feminization.	Kuiper et al. (1998); Kalinina et al. (2017); Vethaak and Legler (2013); Saillenfeit et al. (2016); Muñoz et al. (2023); Kara-Ertekin et al. (2021)

	Androgen receptor (AR) Antagonism	DDT (and its metabolite DDE), Tebuconazole, Vinclozolin, Linuron, Dieldrin, Fenarimol	Competitively binds to the androgen receptor, blocking the action of androgens like dihydrotestosterone (DHT). This can lead to demasculinization, reduced fertility, cryptorchidism, and other reproductive abnormalities.	Kelce et al. (1995); Vethaak & Legler (2013); Lambright (2000); Lv et al. (2017); Danzo (1997)
	Thyroid receptor (TR) Interaction	Chlorpyrifos, DDT, Methoxychlor, Atrazine, Procymidone	Binds directly to thyroid hormone receptors, often exhibiting antagonistic activity. This disrupts thyroid signaling pathways, which are critical for metabolism and neurodevelopment.	Boas et al. (2012); Du et al. (2010); Xiang et al. (2017)
	Aryl hydrocarbon receptor (AhR) modulation	Cypermethrin, Propiconazole, Atrazine	Modulates AhR pathways; affects detoxification and renal function.	Ghisari et al. (2015); Knebel et al. (2018); Zhang et al. (2019).
	Peroxisome proliferator activated receptor (PPAR) modulation	Triflumizole, Diclofop-methyl, Imazalil	Agonistic activity promoting adipogenesis and metabolic disruption.	Takeuchi et al. (2006); Li et al. (2012).
**Non-Genomic Signaling Disruption**	Membrane androgen receptor (ZIP9) antagonism	Prochloraz, Vinclozolin	Inhibits testosterone-induced zinc influx and apoptosis in prostate cancer cells.	Thomas and Dong, (2019).

	Epidermal growth factor receptor (EGFR) pathway inhibition	Atrazine, Cypermethrin	Inhibits EGFR activation and autophosphorylation; suppresses MAPK signaling and cell proliferation.	Hardesty et al. (2018); Wang et al. (2019).
**Disruption of Hormone Synthesis& Metabolism**	Inhibition of steroid hormone synthesis	2,4-D, Atrazine, Cypermethrin, Epoxiconazole	Alters steroidogenic enzyme activity and biosynthetic pathways, decreases testosterone, increases estrogen and cortisol levels, affects cholesterol transport and steroid hormone production.	Harada et al. (2016); Zhang et al. (2017); Háhn et al. (2016); Ji et al. (2019); Lauschke et al. (2021)
	Aromatase inhibition	Atrazine, Flusilazole	Inhibits aromatase (CYP19) activity, reducing estrogen synthesis; linked to feminization.	Vethaak and Legler (2013); Draskau et al. (2021).
	Thyroid hormone synthesis disruption	Amitrole, DDT	Alters thyroglobulin transcription and iodide uptake, disrupting thyroid hormone synthesis and homeostasis.	Pan et al. (2011); Yaglova and Yaglov (2015).
	Hormone metabolism and storage disruption	Fenarimol, Simazine, Thiram, Chlordimeform, Malathion,	Interferes with hormone production, metabolic conversion, and storage processes; affects hormone binding proteins and overall metabolic rate.	Bretveld et al. (2006); Lopez-Antia et al. (2015); Inoue-Choi et al. (2016); Elham et al. (2015).
	Hormone regulation disruption	Lindane, Glyphosate, Malathion, Chlorpyrifos, Dieldrin	Disrupts hormone regulation, affects reproductive and neurological functions; interferes with cholinesterase, neurotransmitter activity, and energy production.	Tiemann (2008); Darbandi et al. (2018); Badr (2020); Lee et al. (2013); Leemans et al. (2019)

	Hormone signaling disruption	DDT analogues, Methoxychlor, Pyrethroids, Diuron, Permethrin.	Alters hormone signaling and metabolism, disrupts reproductive organ development, and affects sodium channels.	Tiemann (2008); Birch et al. (2022); Rahman et al. (2021); Zhou et al. (2022).
**Epigenetic Modifications**	DNA methylation alterations	Atrazine, Vinclozolin, Methyl-parathion, Imidacloprid	Alters DNA methylation patterns in sperm and somatic cells; linked to transgenerational reproductive effects and cancer-related pathways.	McBirney et al. (2017); Nilsson et al. (2018); Hernandez-Cortes et al. (2018); Wang et al. (2021)
	Histone modifications	Atrazine, Endosulfan, Carbendazim, Chlorpyrifos	Modifies histone methylation (e.g., H3K4me3, H3K27me3) and deacetylation; disrupts gene expression linked to reproductive and developmental processes.	Hao et al. (2016); Ghosh et al. (2018); Liu et al. (2019); Ventura et al. (2019)
	Non-coding RNA(miRNA) modulation	Atrazine, DDT	Modifies the expression profile of microRNAs (miRNAs) that regulate key biological processes, including carcinogenesis, cell cycle control, and cellular signaling pathways.	Wirbisky et al. (2016); Tilghman et al. (2012)

**Oxidative stress induction**	Oxidative stress induction	Paraquat, Mancozeb, Cyflumetofen, Epoxiconazole	Generates reactive oxygen species (ROS) and overwhelms cellular antioxidant defenses (e.g., SOD, CAT). This leads to cytotoxicity, protein carbonylation, DNA damage (double-strand breaks), apoptosis, and impaired reproductive function.	Zhou et al. (2022); Dinisri et al. (2021); Zhang et al. (2023); Schwarzbacherová et al. (2017).

Table S2: Epidemiological evidence linking pesticide exposure with reproductive system.

**Study design/ Location**	**Sample characteristics**	**Exposure type/ Biospecimen**	**Pesticide(s)**	**Key findings**	**References**
**Women reproductive disorders**					
Prospective Cohort/ USA	83 women planning a pregnancy	Environmental exposure/Serum	7 OCPs or metabolites	Higher exposure to HCB was associated with a significant reduction in bleeding duration ($\gamma = -0.15$; 95% CI −0.29, −0.00).	Louis et al. (2011)
Case–control/ USA	248 cases /538 controls	Environmental exposure/Serum	7 OCPs or metabolites	Increased risk of ovarian endometriosis with higher β-HCH (highest vs. lowest quartile OR = 1.3; 95% CI: 0.8, 2.4) and mirex (highest vs. lowest quartile: OR = 1.5; 95% CI: 1.0, 2.2) levels.	Upson et al. (2013)
Prospective Cohort/ South Africa	420 women from rural villages	Environmental exposure/ Plasma& Questionnaires	Pyrethroids or DDT	-Women reporting indoor pyrethroid spraying had ~25% lower anti-Müllerian hormone (AMH) concentrations, a marker of ovarian reserve (CI=-39%, -8%).<br>-No clear association was observed between plasma DDT levels and AMH.	Whitworth et al. (2015)
Case–control/ China	50 cases/ 30 controls	Environmental exposure/ Serum	p,p′-DDE<br>HCB<br>β-HCH	Concentrations of p,p′-DDE in serum above median levels were associated with polycystic ovary syndrome OR= 4.89 (95% CI, 1.81–13.2).	Yang et al. (2015)
Cross-sectional/ Kazakhstan	524 adolescent females	Environmental exposure/ peripheral blood	4 OCPs: lindane, dieldrin, DDT, endrin	-A high concentration of OCPs in the blood of females living in rural cotton-growing areas was associated with reduced gonadotropins, estradiol, and IGF-1.	Bapayeva et al. (2016)
Prospective Cohort/USA	32 women undergoing *in vitro* fertilization (IVF).	Environmental exposure/ Ovarian follicular fluid	p,p′-DDT<br>p,p′-DDE	-28% lower likelihoods for retrieval of a mature oocyte among women with higher DDE (RR = 0.72; 95% CI 0.60, 0.86; P = 0.0003).<br>-Women with higher DDT concentrations had a higher average peak estradiol level (β= 1.45; 95% CI 0.92, 1.97; P < 0.0001).	Bloom et al. (2017)

Case-control/China	178 women with polycystic ovary syndrome (PCOS)	Environmental exposure/Serum	p,p-DDT<br>o,p-DDT	-Patients with PCOS had significantly increased serum levels of p,p-DDT ($P = 0.016$) and o,p-DDT ($P = 0.000$).<br>-o,p′-DDT was associated with altered reproductive hormone levels.	Guo et al. (2017)
Case-control/ France	55 cases<br>44 controls	Environmental exposure/Adipose tissue	30 OCPs	Strong associations between multiple OCPs concentrations, including trans nonachlor, cis heptachlor epoxide, dieldrin, and β-HCH and deep infiltrating endometriosis, concurrent with ovarian endometrioma.	Ploteau et al. (2017)
Cross-sectional/ Taiwan	68 woman	Environmental exposure/Breast milk	20 OCPs	-Elevated breast milk levels of γ-HCH were strongly associated with female infertility.<br>-cis-chlordane and γ-HCH were linked to menstrual disturbances, including prolonged (>5 days) or shortened (<3 days) menstrual periods.<br>-Higher levels of 4,4′-DDT were linked to a history of gynecological surgery.	Chen et al. (2018)
Cohort/China	569 women planning a pregnancy	Environmental exposure/Urine	Organophosphate metabolites (DETP) & Pyrethroid metabolite (3-PBA)	4th Q vs. 1st Q DETP and 3-PBA were associated with time-to-pregnancy and infertility in nulliparas.	Hu et al. (2018)
Case-control/China	172 cases /247 control women	Environmental exposure /Urine	Pyrethroid metabolite (3-PBA)	Higher urinary 3-PBA was associated with increased primary ovarian insufficiency risk (OR = 2.344, 95% CI: 1.193–4.607, for the highest vs. lowest quartile), elevated FSH/LH, and reduced anti-Müllerian hormone.	Li et al. (2018)
Case-control/China	157 cases and 217 healthy controls	Environmental exposure/ Serum	15 OCPs	p,p′-DDT was significantly associated with increased primary ovarian insufficiency risk and lower anti-Müllerian hormone levels.	Pan et al. (2019)
Cohort/ Sweden	818 pregnant women	Environmental exposure/Serum	9 OCPs	HCB and p,p′-DDE were associated with infertility and fecundability measured as longer time-to-pregnancy.	Björvang et al. (2020)

Cohort/USA	619 woman	Environmental exposure/Urine	11 pesticide metabolites	A significant association between endometriosis and urinary concentration of diazinon (the parent compound of IMPY; OR = 1.89, 95% Cl = 1.12–3.20) and chlorpyrifos (parent compounds of TCPY; OR = 1.65, 95% Cl = 1.02–2.69).	Li et al. (2020)
Cross-sectional/ Iran	308 greenhouse workers/337 housewives)	Occupational exposure/ Questionnaires	Multiple agricultural pesticides used in greenhouse farming	Greenhouse workers had a significantly greater number of spontaneous pregnancy losses (p = 0.02) and infertility (p = 0.003) than housewives.	Rahimi et al. (2020)
Cross-sectional/ USA	Child and adolescent farmworkers aged 13 to 20 years (n ≈ 98*)	Occupational exposure/ Questionnaires&	Multiple pesticides	Half of participants experienced any menstrual cycle irregularity; the most frequent irregularities were cycle length (38.6%) and having gone 90 days or more without a menstrual period (20.4%).	Varnell et al. (2021)
**Men reproductive disorders**					
Cross-sectional/ Malaysia	152 farmers	Occupational exposure/Semen & Questionnaires	Paraquat & Malathion	Exposed farmers had significantly (p < 0.05) lower semen volume, pH, concentration, and motility and higher sperm abnormality.	Hossain et al. (2010)
Cohort/USA	720 women (mean age 35.4 ± 4.2 year) with *in vitro* fertilization (IVF)	Environmental exposure/Serum	HCB&DDT DDE	-HCB was strongly associated with failed implantation; ORs (vs Q1): Q2 = 1.71 (1.03–2.82), Q3 = 2.30 (1.39–3.81), Q4 = 2.32 (1.38–3.90).<br>-No significant associations were found between DDT or DDE and IVF outcomes.	Mahalingajah et al. (2012)
Case control/USA	16,433 male newborns with genital malformations	Prenatal exposure/ Malformation diagnoses & County-level estimates	Atrazine	Medium to medium-low maternal residential atrazine exposure was consistently associated with increased odds of all male genital malformations, including hypospadias, cryptorchidism, and small penis (e.g., OR ≈ 1.2 for medium vs. low exposure).	Agopian et al. (2013)

Cross-sectional/ Poland	334 men	Environmental exposure/Urine (pyrethroid metabolites); Semen	Pyrethroid Metabolites e.g. 3-PBA	Higher urinary pyrethroid metabolites were associated with increased abnormal sperm morphology and decreased sperm concentration, testosterone levels, and computer-assisted semen analysis parameters.	Radwan et al. (2014)
Cross-sectional/ Greenland, Ukraine	589 men	Environmental exposure/ Serum	HCB	Higher serum HCB levels were positively associated with SHBG and negatively associated with FAI, indicating reduced bioavailable androgens.	Specht et al. (2015)
Cross-sectional/ Brazil	99 rural & 36 urban men (18–23 years).	Occupational exposure/ Serum & semen & Questionnaires	Multiple pesticides, such as herbicides, fungicides, pyrethroids, and insecticides	-Lifetime use of pesticides for rural men, especially herbicides and fungicides, was associated with poorer semen morphology and reduced LH and prolactin, with evidence of a linear pattern.<br>-Maternal farming during pregnancy was associated with larger anogenital distance and testis volume.	Cremonese et al. (2017)
Cross-sectional/South Africa	535 men (18-40 years)	Environmental exposure/Plasma	DDT, DDE	Men with high DDE, DDT concentrations had free and bio-available testosterone and estradiol E, but low FSH, LH concentrations.	Bornman et al. (2018)
Cohort/ Denmark	420 boys examined 3 months after expected date of delivery	Prenatal exposure/Maternal urine during gestation (gw 28)	2,4-D	Maternal 2,4-D exposure (second vs first tertile) associated with significantly shorter anogenital distance (β: −1.55 (-2.81, −0.28) mm).	Dalsager et al. (2018)
Case-control/ France	25 case/58 control	Prenatal exposure/ Meconium	11 pesticides and metabolites	-Isoproturon (OR= 5.94 CI: 1.03–34.11) and MCPA (OR=4.75 CI: 1.20–18.76) were associated with isolated hypospadias in the newborns.	Haraux et al. (2018)

Cohort/ Mexico	74 boys	Prenatal exposure /Maternal serum during third trimester of pregnancy	p,p′-DDE	A significant positive association was observed between anogenital distance, the distance from the center of the anus to the anterior base of the penis relative to height, and maternal concentrations of p,p′-DDE.	Loreto-Gómez et al. (2018)
Cross-sectional/ Myanmar	100 groundnut farmers	Occupational exposure/Blood& semen& Questionnaires for pesticides exposure	Ops used in the season	Statistically significant differences (P<0.05) in seminal parameters (pH, viscosity, count, motility, and sperm morphology) and in a reproductive hormonal assay (FSH and testosterone) between the growing and nongrowing periods.	Lwin et al. (2018)
Cross-sectional/ Poland	315 men (<45 years)	Environmental exposure/ Urine (exposure); semen (outcome)	2 metabolites; (1N) & (TCPY) of nonpersistent insecticides	-TCPY concentration was associated with decreased motility and a positive association with the DNA fragmentation index.<br>-1N negatively associated with normal morphology and positively with computer-aided semen analysis (CASA).	Dziewirska et al. (2019)
Cross-sectional/China	346 men	Environmental/ Urine (Pyrethroids metabolites); semen samples	Pyrethroids metabolites: (3-PBA & TDCCA & CDCCA)	-Higher 3-PBA levels were negatively associated with sperm morphology, and higher TDCCA levels with lower total sperm count.<br>-Men in the highest quartile of 3-PBA had increased odds of poor semen quality and abnormal morphology.	Hu et al. (2020)
Case-cohort/ Norway	641 male infants	Perinatal exposure/Breast milk	β-HCH	β-HCH was associated with increased odds of cryptorchidism (OR = 1.26, 95% CI: 1.03–1.53).	Desalegn et al. (2021)
Retrospective /France	671 men	Occupational or environmental exposure/ Self-reported exposure & Semen	Self-reported exposure to pesticides (mixed/unspecified classes)	-A significant decline in sperm vitality, as well as progressive and non-progressive motility, was observed in the pesticide-exposed group.<br>-Higher levels of DNA fragmentation and chromatin decondensation were found in the pesticide-exposed group compared to the non-exposed group.	Lahimer et al. (2023)

Cross-sectional/ /Switzerland	2326 Swiss conscripts	Occupational exposure/ Serum & Questionnaire	Multiple pesticides (Occupational exposure)	Maternal occupational exposure to pesticides was significantly associated with higher sex hormone–binding globulin (SHBG) levels in adult sons.	Blanc-Petitjean et al. (2026)
Cross-sectional/ China	103 men	Environmental exposure/ Plasma	p,p′-DDE Bifenthrin	p,p′-DDE levels were negatively associated with sperm viability.	Wang et al. (2025)

OCPs: organochlorine pesticides; HCB: Hexachlorobenzene; β-HCH: β-hexachlorocyclohexane; DDE: dichlorodiphenylethylene; DDT: dichlorodiphenyltrichloroethane; p,p'-DDE: p,p′-Dichlorodiphenyldichloroethylene; IGF-1: insulin-like growth factor; p,p'-DDT: p,p′-Dichlorodiphenyltrichloroethane; DETP: diethylthiophosphate; 3-PBA: 3-phenoxybenzoic acid; SHBG: Sex Hormone Binding Globulin; FAI: Free Androgen Index; 2,4-D: 2,4-Dichlorophenoxyacetic acid; FSH: Follicle-stimulating hormone; TCPY: 3,5,6-trichloro-2-pyridinol; TDCCA: trans -3-(2,2-Dichlorovinyl) −2,2-dimethylcy clopropane carboxylic acid; CDCCA: Cis-3-(2,2-Dichlorovinyl) −2,2-dimethylcy clopropane carboxylic acid

Table S3: Epidemiological evidence linking pesticide exposure with thyroid toxic effects.

**Study design/ Location**	**Sample characteristics**	**Exposure type/ Biospecimen**	**Pesticide(s)**	**Key findings**	**References**
Cohort/USA	36357 applicators reported using atrazine	Occupational exposure/ Questionnaire	Atrazine	A significantly elevated risk of thyroid cancer was observed in the 2nd (RR=4.55, 1.27-16.24) and 4th (RR=4.84, 1.31-17.93) quartiles of intensity-weighted lifetime days.	Freeman et al. (2011)
Cohort/USA	29325 female spouses of pesticide applicators	Occupational exposure/ Self-reported personal use of OPs	10 OPs	-Malathion was associated with increased thyroid cancer risk (RR = 2.04; 95% CI: 1.14–3.63).	Lerro et al. (2015)
Longitudinal study /Mexico	136 male floricultural workers	Occupational exposure/ Serum	p,p'-DDE	-Serum *p,p′*-DDE levels are associated with an increase of both total T3 (β=0.01, 95% CI: −0.009, 0.03) and total T4 (β=0.08, 95% CI: 0.03, 0.14) levels.<br>-Serum *p,p′*-DDE levels are associated with a non significant decrease in TSH levels.	Blanco-Muñoz et al. (2016)
Cross-sectional/ Brazil	275 male and female farm residents	Occupational exposure/ Questionnaires & Serum	Fungicides, herbicides, and dithiocarbamates	In men, lifetime use of fungicides, herbicides, and dithiocarbamates was associated with ↑ TSH and ↓ free T4.<br>- In women, no consistent associations with thyroid hormones.	Piccoli et al. (2016)
Cross-sectional/China	115 newborns	Prenatal exposure/Cord blood	17 OCPs	- Lower FT4 was associated with higher cord plasma levels of ∑HCHs, ϱ,ϱ′-DDE, and methoxychlor, but not with free T3 and TSH levels.<br>- Higher TSH levels were linked to higher amounts of aldrin, dieldrin, and ∑DDTs in cord plasma after adjustment for confounders.	Luo et al. (2017)

Case-control/USA	462 incident thyroid cancer cases/ 498 controls	Occupational exposure/Questionnaire	Biocides	Individuals who were occupationally exposed to biocides had an increased risk of thyroid cancer (OR=1.65, 95% CI 1.16 to 2.35).	Zeng et al. (2017)
Cohort/USA	24,092 female spouses	Occupational/Self-reported doctor-diagnosed thyroid disease	Multiple pesticides, including fungicides, insecticides, and herbicides	-For hypothyroidism, several individual pesticides were associated with increased risk (HR ranging from 1.56 to 2.44), including benomyl, maneb/mancozeb, metalaxyl, and pendimethalin.<br>-For hyperthyroidism, several pesticides were associated with increased risk (HR ranging from 1.35 to 2.01), including diazinon, mancozeb, and metolachlor.	Shrestha et al. (2018)
Cohort/USA	679 male licensed pesticide applicators	Occupational/Serum	Aldrin, Pendimethalin, Methyl bromide	-Aldrin:↑risk of hypothyroidism (OR≈4), ↑TSH, ↓T4.<br>-Pendimethalin: ↑ risk of hypothyroidism (OR≈2.8), ↑TSH, ↑anti- antithyroid peroxidase.<br>-Methyl bromide: ↓TSH, ↑T4.	Lerro et al. (2018)
Case-control/China	168: diagnosed with thyroid disease/ 186 without thyroid disease	Environmental/ Structured interview and blood sampling	Multiple OCPs	Exposure to OCPs was significantly and positively associated with thyroid cancer in Chinese adults (OR= 1.97, 1.07–3.63).	Han et al. (2019)
Case-control/Spain	79.431 individuals diagnosed with goiter, thyrotoxicosis, hypothyroidism, and thyroiditis/ 1.484.257 controls	Environmental exposure/ Computerized records of the Public Health Service of Andalusia	multiple pesticide classes; areas categorized as of high or low pesticide use	Populations living in high pesticide-use areas had significantly increased risks of thyroid disease:<br>-49% risk of hypothyroidism<br>-45% risk of thyrotoxicosis<br>-20% risk of thyroiditis<br>-5% risk of goiter	Requena et al. (2019)
Case-Control/ Belgium	239 individuals	Environmental exposure/Serum	16 OCPs	p,p'-DDE was associated with an increased risk of several thyroid conditions: hypothyroidism (OR=	Dufour et al. (2020)

				4.47, CI: 0.96-20.8), and any autoimmune thyroid disease (OR = 3.08, CI: 1.02–9.35).	
Cohort/ Japan	333 mothers and infant pairs	Prenatal exposure/ Maternal blood during early pregnancy	29 OCPs	• Higher o,p′-DDE, o,p′-DDT, and dieldrin were associated with lower maternal FT4.<br>Cis-nonachlor and mirex were positively associated with infant FT4	Yamazaki et al. (2020)
Cohort/USA	53096 licensed male pesticide applicators	Occupational exposure/ Questionnaire	Participants reported use of 50 pesticides 50 pesticide	-Metalaxyl (HR = 2.03, CI:1.16–3.52) and lindane (HR = 1.74, CI:1.06–2.84) were associated with increased thyroid cancer risk among male applicators.<br>-Carbaryl showed inverse associations (HR = 0.20, 0.08–0.53).	Lerro et al. (2021)
Case-control/USA	2067 cases and 1003 controls ≥35 years of age	Environmental exposure/ Interview	29 agricultural use pesticides	10 out of 29 selected pesticides were associated with thyroid cancer, such as paraquat dichloride [OR: 1.46 (95% CI: 1.23, 1.73)], glyphosate [OR: 1.33 (95% CI: 1.12, 1.58)], and oxyfluorfen [OR: 1.21 (95% CI: 1.02, 1.43)].	Omidakhsh et al. (2022)
Cross-sectional/ China	1054 >60 years	Environmental exposure/ Serum	19 OCPs, 23 OPs, 8 synthetic pyrethroids, and 18 herbicides	-1 OP (dichlorvos) and 1 herbicide (propham) were negatively associated with fT4.<br>-4 OCPs were negatively associated with tT4.<br>-2 herbicides (benoxacor and acetochlor) and 2 synthetic pyrethroids (permethrin and fenvalerate) were positively associated with fT3 (all $P<0.05$).	Han et al. (2024)
Case-control/USA	742 cases with papillary thyroid Cancer (PTC)/742 control	Environmental exposure/ Serum	9 OCPs	Classical PTC was associated with increased concentrations of HCB and β-HCCH, which were stronger among females.	Rusiecki et al. (2024)
Retrospective cross-sectional/China	3425 participants	Environmental exposure/Urine	TCPy/ chlorpyrifos metabolite &PNP/ parathion metabolite	-Both PNP and TCPy showed significant positive associations with subclinical hyperthyroidism.	Wei et al. (2025)

Cross-sectional/ China	2187 school-aged children (8–11 years)	Environmental exposure /Urine	Pesticide metabolites (organophosphate, pyrethroid, and phenoxy carboxylic acid)	-Risk assessment revealed 0.89 % of children were at risk from parathion exposure.<br>-Pyrethroid metabolites showed negative associations with thyroid volume.	Zhong et al. (2025)

OCPs: Organochlorine pesticides; OPs: Organophosphate insecticides; RR: Relative risks; p,p'-DDE: p,p′-dichlorodiphenyldichloroethylene; TSH: Thyroid-stimulating hormone; HR: Hazard ratios; T_3: Triiodothyronine T_4: Thyroxine; ∑DDT: ∑Dichlorodiphenyltrichloroethane; OR: Odds ratio; CI: Confidence interval; tT4: Total thyroxine. fT4: Free thyroxine; fT3: Free triiodothyronine. HCB: hexachlorobenzene; β-HCCH: β-hexachlorocyclohexane; TCPy: 3,5,6-trichloropyridinol; PNP: paranitrophenol

References (Supplementary Data):

Badr, A. M. (2020). Organophosphate toxicity: updates of malathion potential toxic effects in mammals and potential treatments. *Environmental science and pollution research*, *27*(21), 26036-26057. doi: 10.1007/s11356-020-08937-4.

Bloom, M. S., Fujimoto, V. Y., Storm, R., Zhang, L., Butts, C. D., Sollohub, D., & Jansing, R. L. (2017). Persistent organic pollutants (POPs) in human follicular fluid and in vitro fertilization outcomes, a pilot study. *Reproductive Toxicology*, *67*, 165-173. https://doi.org/10.1016/j.reprotox.2017.01.004

Chen, M. W., Santos, H. M., Que, D. E., Gou, Y. Y., Tayo, L. L., Hsu, Y. C., ... & Huang, K. L. (2018). Association between organochlorine pesticide levels in breast milk and their effects on female reproduction in a Taiwanese population. *International Journal of Environmental Research and Public Health*, *15*(5), 931. https://doi.org/10.3390/ijerph15050931

Danzo, B. J. (1997). Environmental xenobiotics may disrupt normal endocrine function by interfering with the binding of physiological ligands to steroid receptors and binding proteins. *Environmental health perspectives*, *105*(3), 294. doi: 10.1289/ehp.97105294

Dufour, P., Pirard, C., Petrossians, P., Beckers, A., & Charlier, C. (2020). Association between mixture of persistent organic pollutants and thyroid pathologies in a Belgian population. *Environmental research*, *181*, 108922. https:// doi.org/ 10. 1016/j. envres. 2019. 108922.

Han, X., Meng, L., Li, Y., Li, A., Turyk, M. E., Yang, R., ... & Jiang, G. (2019). Associations between exposure to persistent organic pollutants and thyroid function in a case-control study of East China. *Environmental Science & Technology*, *53*(16), 9866-9875. DOI: 10.1021/acs.est.9b02810

Kalinina, T. S., Kononchuk, V. V., & Gulyaeva, L. F. (2017). Expression of hormonal carcinogenesis genes and related regulatory microRNAs in uterus and ovaries of DDT-treated female rats. *Biochemistry (Moscow)*, *82*(10), 1118-1128. doi: 10.1134/S0006297917100042.

Kara-Ertekin, S., Yazar, S., & Erkan, M. (2021). In vitro toxicological assessment of flumethrin's effects on MCF-7 breast cancer cells. *Human & Experimental Toxicology*, 40(12), 2165-2177. https://doi.org/ 10.1177/09603271211022789

Knebel, C., Kebben, J., Eberini, I., Palazzolo, L., Hammer, H. S., Süssmuth, R. D., ... & Marx-Stoelting, P. (2018). Propiconazole is an activator of AHR and causes concentration additive effects with an established AHR ligand. *Archives of toxicology*, *92*(12), 3471-3486. doi: 10.1007/s00204-018-2321-x.

Leemans, M., Couderq, S., Demeneix, B., & Fini, J. B. (2019). Pesticides with potential thyroid hormone-disrupting effects: a review of recent data. *Frontiers in endocrinology*, *10*, 743. https://doi.org/10.3389/fendo.2019.00743

Luo, D., Pu, Y., Tian, H., Wu, W., Sun, X., Zhou, T., ... & Mei, S. (2017). Association of in utero exposure to organochlorine pesticides with thyroid hormone levels in cord blood of newborns. *Environmental Pollution*, *231*, 78-86. https://doi.org/10.1016/j.envpol.2017.07.091

Lwin, T. Z., Than, A. A., Min, A. Z., Robson, M. G., & Siriwong, W. (2018). Effects of pesticide exposure on reproductivity of male groundnut farmers in Kyauk Kan village, Nyaung-U, Mandalay region, Myanmar. *Risk management and healthcare policy*, 235-241. https://doi.org/10.2147/RMHP.S175230

Mahalingaiah, S., Missmer, S. A., Maity, A., Williams, P. L., Meeker, J. D., Berry, K., ... & Hauser, R. (2012). Association of hexachlorobenzene (HCB), dichlorodiphenyltrichloroethane (DDT), and dichlorodiphenyldichloroethylene (DDE) with *in vitro* fertilization (IVF) outcomes. *Environmental health perspectives*, *120*(2), 316. doi: 10.1289/ehp.1103696

Omidakhsh, N., Heck, J. E., Cockburn, M., Ling, C., Hershman, J. M., & Harari, A. (2022). Thyroid cancer and pesticide use in a central California agricultural area: a case control study. *The Journal of Clinical Endocrinology & Metabolism*, *107*(9), e3574-e3582. https://doi.org/10.1210/clinem/dgac413

Rahman, H. U., Asghar, W., Nazir, W., Sandhu, M. A., Ahmed, A., & Khalid, N. (2021). A comprehensive review on chlorpyrifos toxicity with special reference to endocrine disruption: Evidence of mechanisms, exposures and mitigation strategies. *Science of The Total Environment*, *755*, 142649. https://doi.org/10.1016/j.scitotenv.2020.142649

Saillenfait, A. M., Ndiaye, D., & Sabaté, J. P. (2016). The estrogenic and androgenic potential of pyrethroids in vitro. Review. *Toxicology in Vitro*, *34*, 321-332. https://doi.org/10.1016/j.tiv.2016.02.020

Shrestha, S., Parks, C. G., Goldner, W. S., Kamel, F., Umbach, D. M., Ward, M. H., ... & Sandler, D. P. (2018). Incident thyroid disease in female spouses of private pesticide applicators. *Environment international*, *118*, 282-292. https://doi.org/10.1016/j.envint.2018.05.041

Ventura, C., Zappia, C. D., Lasagna, M., Pavicic, W., Richard, S., Bolzan, A. D., ... & Cocca, C. (2019). Effects of the pesticide chlorpyrifos on breast cancer disease. Implication of epigenetic mechanisms. *The Journal of steroid biochemistry and molecular biology*, *186*, 96-104. https://doi.org/10.1016/j.jsbmb.2018.09.021

Wang, Z., Fu, K., Zhang, X., Chen, G., Zheng, L., Liang, Y., ... & Hong, H. (2025). Plasma exposure levels of polycyclic aromatic hydrocarbons, pesticides and metals/metalloids among reproductive-age men in Jinhua, China: Influencing factors and links to semen quality. *Ecotoxicology and Environmental Safety*, *305*, 119191. https://doi.org/10.1016/j.ecoenv.2025.119191

Zeng, F., Lerro, C., Lavoué, J., Huang, H., Siemiatycki, J., Zhao, N., ... & Zhang, Y. (2017). Occupational exposure to pesticides and other biocides and risk of thyroid cancer. *Occupational and environmental medicine*, *74*(7), 502-510. https://doi.org/10.1136/oemed-2016-103931
